# Supplementary material for: Diversity, taxonomy, and evolution of archaeal viruses of the class Caudoviricetes
Source: PLoS Biol. 2021 Nov 9;19(11):e3001442. doi: 10.1371/journal.pbio.3001442 (PMC8651126; doi:10.1371/journal.pbio.3001442)
Supplement: S1 Text — (PDF) [file pbio.3001442.s001.pdf]

SUPPLEMENTARY TEXT

Description of the proposed virus families

Clade I in the GRAViTy analysis consists of four family-level groups (Fig 1A). Among these, family (F) 1 is the largest, with 39 members, which can be further divided into four genus-level (G) subgroups (Fig 2). F1G1 (virus cluster [VC] 77) consists of 31 viruses, including 25 newly sequenced viruses as well as the previously reported viruses HF1, HF2, HRTV-5, HRTV-8, Serpecor1 and Hardycor2 (Fig 1B, S1 Table) . Using the 95% nucleotide sequence identity threshold as a species demarcation criterion [1], the 31 viruses were assigned to seven species. F1G2 (VC_80) consists of a single species, including six closely related viruses, namely, new viruses HRTV-2, HRTV-11, HCTV-6 and HCTV-15 as well as the previously reported HSTV-2 and HRTV-7. F1G3 and F1G4 each contains a single new virus, HRTV-25 and HRTV-27, respectively, which were identified as outliers tightly linked to the other F1 virus genomes (Fig 2). The viruses from the four genera in F1 share the virion orphogenesis and genome replication modules. The morphogenetic module consists of 18 orthologous genes responsible for the formation of virions with myovirus morphology, whereas replication module includes family B DNA polymerase (PolB), archaeo-eukaryotic primase (AEP) and replicative minichromosome maintenance (MCM) helicase (S1A Fig). The genus-specific genes constitute almost half of the gene contents and are located mainly in the right arm of the genome, highlighting the fluidity of this region (S1A Fig).

The F2 in Clade I consists of 5 members divided into two genera, consistent with the network analysis (Fig 1, Fig 2). F2G1 (VC_226_1) includes HVTV-2, HVTV-1 and HCTV-5, whereas F2G2 (VC_226_0) includes HCTV-16 and HCTV-1. Viruses in the two genera contain largely syntenic genomes, but differ within the ~14 kb-long tail module (S1B Fig). Notably, viruses from F2G1 (but not F2G2) share most of the tail genes with viruses from F11 (HCTV-2 and HHTV-2), despite the fact that F11 viruses have genomes only half the size of those in F2. Most likely, the tail module has been acquired by the ancestor of HVTV-1 through recombination from a F11 member following its divergence from the common ancestor with the HCTV-1 (S1B Fig). Consistent with this possibility, viruses from F2 and F11 infect *Haloarcula* strains.

HATV-2 and HGTV-1 are singletons in F3 and F4, respectively (Fig 1). In the network analysis, the two genomes formed a single cluster, VC_66, albeit with a low topology confidence score (Fig 2, spreadsheet “Fig 2 VCs” in S2 Data). However, given the difference in genome sizes (HGTV-1 is more than twice larger than HATV-2; S1C Fig), different mechanisms of genome packaging (HATV-2 has direct terminal repeats, whereas HGTV-1 uses the headful mechanism (S1 Table)), we propose classifying them into separate families. The two viruses share 12 genes (~4% and ~10% of the HGTV-1 and HATV-2 genomes, respectively), including those for several structural proteins and genome replication proteins (S1C Fig).

More generally, Clade I is a cohesive assemblage held together by 34 protein clusters (PCs), including those involved in virion morphogenesis (baseplate wedge J-like protein, tail sheath, prohead protease), genome replication (PolB and MCM), nucleotide metabolism/DNA repair (thymidylate synthase thyX, dUTPase, thymidylate kinase, DNA methyltransferase [MTase], ribonucleotide diphosphate reductase [RnR], dCTP tRNA splicing ligase RtcB, holiday junction resolvase), and several proteins of unclear functions, such as SprT-like metalloprotease, dual specificity protein phosphatase (DUSP), various nucleases and proteins with SPFH and ATPase domains (S2 Table). Thus, the four families from Clade I can be unified into a virus order, *Thumleimavirales*, within the class *Caudoviricetes*.

Clade II consists of 10 family-level groupings, half of which consist of singletons (Fig 1A and 1C). F5 contains three genera represented by viruses HRTV-29, HFTV1 and HRTV-4, respectively (Fig 1C, Fig 2). Pairwise genomic comparison showed an overall similar genomic organization of the three viruses, including the genes in the morphogenesis module. The

replication related genes, however, are distinct in these viruses: HFTV1 encodes MCM and PCNA, HRTV-29 has MCM only, whereas HRTV-4 has neither of the two (S1D Fig).

Two newly sequenced viruses, HATV-3 and HRTV-28, as well as the previously described HSTV-1 are singletons in the F6, F7 and F10, respectively, and are connected to members of the F5 as outliers in the network (Fig 2). The three viruses share only a handful of genes with each other as well as with HFTV1 from F5 (S1E Fig). These include genes involved in virion morphogenesis (TerS, portal, MCP and baseplate hub), genome replication (PCNA and MCM), and other functions (Rad52, HNH and GIY-YIG endonucleases etc.) (S1E Fig, S2 Table).

The F8 consists of two viruses, BJ1 and CGphi46, and they are sufficiently distinct to be classified into separate genera. Although the two viruses formed a single cluster (VC_255) in the network analysis (Fig 2), comparative genomics revealed that more than half of the genes in the two viruses are unrelated (S1F Fig). In the two viruses, all genes in the structural module are conserved, except for *terL*, while genes involved in their genome replication are distinct, with Orc1/Cdc6 and MCM encoded by BJ1, whereas a primase-helicase and PCNA are encoded by CGphi46 (S1F Fig).

The F9 contains two genera, with phiCh1 and phiH1 forming one genus, and ChaoS9 forming the other one. Although the three viruses formed a single cluster in the network analysis (Fig 2), the genome alignments showed that ChaoS9 shares with the other two viruses only the tail morphogenesis module (~1/3 of the genome; S1G Fig). Therefore, we propose extracting ChaoS9 from the previously proposed genus *Myohalovirus* [2] and assigning it into a separate genus.

The F11 contains two single-species genera, represented by viruses HCTV-2 and HHTV-2, respectively. In the vContact analysis the two viruses are classified into a single cluster (VC_249), but due to low fraction of shared genes (Fig 3B, S2 Fig), they were classified into separate genera. As mentioned above, HCTV-2 and HHTV-2 are connected to viruses in F2G1 in the network analysis due to the shared tail proteins (Fig 2, Fig 1C). F12 contains a previously described virus HHTV-1, which was identified as a singleton in the network analysis, although it shares several proteins (e.g., TerS, MCP, tail tape measure protein [TMP], PCNA, etc.) with other archaeal tailed viruses from distinct families (S2 Table).

F13 and F14 include tailed viruses infecting methanogenic archaea. F13 includes virus psiM2, which is related to the previously reported defective provirus psiM100 (VC_287) (Fig 2), but shows no appreciable sequence similarity to haloarchaeal tailed viruses. F14 also includes a single representative, virus Drs3. The two viruses of methanogens share TerL, portal, prohead protease and MCP (S1H Fig). Although identified as the singleton in the network analysis, Drs3 shares several proteins with the haloarchaeal relatives, including the prohead protease, Mu gpF-like protein, DNA methyltransferase (MTase) and ERCC4 nuclease (S2 Table).

Defense and counterdefense factors

Restriction-modification (RM) systems and their stand-alone components represent the most common potential mechanisms of defense and counterdefense employed by archaeal tailed viruses. Forty-eight out of 63 viruses from 10 families encode MTases, presumably to methylate the viral genome to escape restriction by the host RM systems. One viral genome can encode up to four MTases, which can methylate N6-adenine, N4-cytosine and C5-cytosine (S4 Table, S3 Fig). The N6-adenine Dam MTase encoded by phiCh1 has been shown to methylate viral genome at G^{m6}ATC and related sequences [3]. The homologs of phiCh1 MTase are encoded in three other viruses, namely, phiH1, HRTV-27 and HRTV-28. Notably, the MTase recognition motif, GATC, shows high frequency (6.47-11.04/kb) in these genomes. In contrast, the same motif is absent from genomes of viruses of the *Hafunaviridae* (except HRTV-27), *Druskaviridae*, *Soleiviridae*, *Halomagnusviridae* and *Saparoviridae*, none of which

encodes Dam MTases. HRTV-27 is the only member of the *Hafunaviridae* which encodes a Dam MTase and, accordingly, contains the GATC tetramer. This indicates that archaeal tailed viruses have likely evolved to escape host RM system either by self-methylation by different virus-encoded MTases or by purging the recognition motifs from their genomes.

In addition to stand-alone MTases, some archaeal viruses encode accompanying restriction endonucleases (REases), forming apparently functional RM systems or stand-alone nucleases. Four viruses, HRTV-17, phiH1, HCTV-2 and HHTV-2, encode micrococcal nucleases (S3 Table), which hydrolyze single- or double- stranded DNA and RNA [4]. The latter are likely to be functional, given that the Ca^{2+}-binding and catalytic sites are conserved (S4 Fig). HJTV-2 and HGTV-1 encode type II REases Eco29KI and EcoRV, which recognize "CCGCGG" and "GATATC" sequences, respectively (S3 Table). Notably, although at least HJTV-2 encodes the cognate MTase, both "CCGCGG" and "GATATC" motifs are absent from the corresponding virus genomes. Analysis of the phyletic distribution of the two REases suggests that both genes (along with the cognate MTases) have been horizontally introduced into the haloarchaeal virus genomes from bacteria. These observations suggest that, similar to tailed bacteriophages, haloarchaeal tailed viruses encode REases/RM systems for degradation of the host chromosomes or genomes of competing mobile genetic elements.

Many bacteriophages and some hyperthermophilic archaeal viruses encode diverse anti-CRISPR (Acr) proteins, which block the CRISPR response by different mechanisms [5]. Most Acr proteins are virus-specific, but several protein families are broadly distributed in bacterial and archaeal viruses. Virus-encoded homologs of the Cas4 nuclease represent one of such potential Acr protein families widespread in viral genomes [6-8]. Viruses of the families *Hafunaviridae* and *Druskaviridae* encode putative Cas4-like nucleases containing the conserved residues involved in the coordination of the Fe-S cluster as well as the conserved RecB-like exonuclease domain [9]. It is possible that these Cas4 nucleases function in counterdefense against the haloarchaeal CRISPR-Cas systems. Furthermore, there are many candidates for potential Acr proteins among the haloarchaeal virus genes lacking functional annotation, but experimental studies are necessary to reveal their function.

Tail fiber proteins of haloarchaeal head-tail viruses

Multiple sequence alignment of the glycine-rich proteins encoded by hafunaviruses revealed six to seven hypervariable segments (HVSs) interspersed by glycine-rich motifs (GRMs) (S8A Fig), a feature common with adhesins located at the distal tip of the tail fibers of diverse T-even phages [10, 11]. The HVSs in phage adhesins have been shown to determine the host specificity [12]. Indeed, one of the two mutations identified in HRTV-19 and HRTV-23 results in a single substitution (A217V) within HVS4 (S9 Fig). The adhesins encoded by hafunaviruses form four distinct phylogenetic groups, with proteins in Groups 1, 2 and 4 having short HVSs and those in Group 3 containing long HVSs (S9 Fig). Comparison of the host ranges of viruses from each adhesin group showed that viruses encoding Group 1 adhesins with short HVSs , the extent of sequence divergence between adhesins corresponds to the differences in the host ranges, i.e., the more similar a pair of adhesins is, the more similar are the host ranges of the corresponding viruses. By contrast, viruses encoding Group 3 adhesins with long HVSs displayed more variable host ranges that did not always match the divergence of the corresponding adhesins. With only two viruses tested in the study that encode Group 2 and Group 4 adhesins, respectively, the features of their adhesin−host range relationship are not clear (S9 Fig).

Analysis of the gene content and organization of the tail morphogenesis modules suggests that the VP1-like minor structural protein, encoded between the adhesin and the P2 gpI-like baseplate protein genes (S1A Fig, S7 Fig) is likely the main component of the tail fiber that bridges the adhesin to the baseplate. Notably, phylogenetic analysis of VP1-like proteins shows that these proteins do not (necessarily) co-evolve with the adhesins (S10 Fig),

suggesting that the genes encoding the two components of the tail fiber are exchanged by recombination between haloarchaeal tailed viruses. Thus, our analysis suggests that the broad host range of hafunaviruses is an additive effect of the fast evolution and existence of several groups of adhesins, combined with their frequent recombination with VP1-like genes . The ability to rapidly explore the host specificity landscape appears to be critical in hypersaline environments, where virus-to-cell ratios exceed those in many other environments [13, 14].

Structural comparison of the arTV major capsid proteins

To gain further insight into the divergence of the arTV MCPs, we performed structural modeling using AlphaFold2 [15] and RoseTTAFold [16] for representatives of all 14 arTV families as well as selected uncultured arTVs (Fig 7A). The structural models were then compared to each other in all-against-all analysis (Fig 7B) and a cladogram was derived from pairwise structural similarity (Z) scores (Fig 7A). This analysis confirmed that all identified arTV MCPs have the canonical HK97 structural fold, shared with tailed bacteriophages and eukaryotic herpesviruses, and revealed the same nine MCP clades obtained using sequence-based phylogenetic analysis (Fig 6, Fig 7). Besides the subtle structural differences among different arTVs, the more pronounced variation was observed in the N-termini of certain MCPs. In particular, HATV-3, HCTV-2, HFTV1, HRTV-28, HSTV-1 and HVTV-1 contained N-terminal 100-120 aa extensions (not shown in Fig. 7A), equivalent to the scaffolding Δ-domain of the HK97 MCP, which is essential for capsid assembly and is cleaved from the mature MCP [17].

SUPPLEMENTARY REFERENCES

1. Krupovic M, Dutilh BE, Adriaenssens EM, Wittmann J, Vogensen FK, Sullivan MB, et al. Taxonomy of prokaryotic viruses: update from the ICTV bacterial and archaeal viruses subcommittee. Archives of virology. 2016;161(4):1095-9. Epub 2016/01/07. doi: 10.1007/s00705-015-2728-0. PubMed PMID: 26733293;
2. Dyall-Smith M, Palm P, Wanner G, Witte A, Oesterhelt D, Pfeiffer F. Halobacterium salinarum virus ChaoS9, a Novel Halovirus Related to PhiH1 and PhiCh1. Genes (Basel). 2019;10(3). Epub 2019/03/06. doi: 10.3390/genes10030194. PubMed PMID: 30832293; PMC6471424.
3. Baranyi U, Klein R, Lubitz W, Kruger DH, Witte A. The archaeal halophilic virus-encoded Dam-like methyltransferase M. phiCh1-I methylates adenine residues and complements dam mutants in the low salt environment of Escherichia coli. Molecular microbiology. 2000;35(5):1168-79. Epub 2000/03/11. doi: 10.1046/j.1365-2958.2000.01786.x. PubMed PMID: 10712697;
4. Ponting CP. P100, a transcriptional coactivator, is a human homologue of staphylococcal nuclease. Protein Sci. 1997;6(2):459-63. Epub 1997/02/01. doi: 10.1002/pro.5560060224. PubMed PMID: 9041650; PMC2143632.
5. Li Y, Bondy-Denomy J. Anti-CRISPRs go viral: the infection biology of CRISPR-Cas inhibitors. Cell host & microbe. 2020. Epub 2021/01/15. doi: 10.1016/j.chom.2020.12.007. PubMed PMID: 33444542;
6. Hudaiberdiev S, Shmakov S, Wolf YI, Terns MP, Makarova KS, Koonin EV. Phylogenomics of Cas4 family nucleases. BMC Evol Biol. 2017;17(1):232. Epub 2017/11/29. doi: 10.1186/s12862-017-1081-1. PubMed PMID: 29179671; PMC5704561.
7. Zhang Z, Pan S, Liu T, Li Y, Peng N. Cas4 Nucleases Can Effect Specific Integration of CRISPR Spacers. Journal of bacteriology. 2019;201(12). Epub 2019/04/03. doi: 10.1128/JB.00747-18. PubMed PMID: 30936372; PMC6531622.
8. Hooton SP, Connerton IF. Campylobacter jejuni acquire new host-derived CRISPR spacers when in association with bacteriophages harboring a CRISPR-like Cas4 protein. Front Microbiol. 2014;5:744. Epub 2015/01/21. doi: 10.3389/fmicb.2014.00744. PubMed PMID: 25601859; PMC4283603.
9. Senčilo A, Jacobs-Sera D, Russell DA, Ko CC, Bowman CA, Atanasova NS, et al. Snapshot of haloarchaeal tailed virus genomes. RNA biology. 2013;10(5):803-16. Epub

2013/03/09. doi: 10.4161/rna.24045. PubMed PMID: 23470522; PMC3737338.
10. Dyall-Smith M, Tang SL, Russ B, Chiang PW, Pfeiffer F. Comparative genomics of two new HF1-like haloviruses. Genes (Basel). 2020;11(4):405. Epub 2020/04/12. doi: 10.3390/genes11040405. PubMed PMID: 32276506; PMC7230728.
11. Dunne M, Denyes JM, Arndt H, Loessner MJ, Leiman PG, Klumpp J. Salmonella Phage S16 Tail Fiber Adhesin Features a Rare Polyglycine Rich Domain for Host Recognition. Structure (London, England : 1993). 2018;26(12):1573-82 e4. Epub 2018/09/25. doi: 10.1016/j.str.2018.07.017. PubMed PMID: 30244968;
12. Trojet SN, Caumont-Sarcos A, Perrody E, Comeau AM, Krisch HM. The gp38 adhesins of the T4 superfamily: a complex modular determinant of the phage's host specificity. Genome Biol Evol. 2011;3:674-86. Epub 2011/07/13. doi: 10.1093/gbe/evr059. PubMed PMID: 21746838; PMC3157838.
13. Sime-Ngando T, Lucas S, Robin A, Tucker KP, Colombet J, Bettarel Y, et al. Diversity of virus-host systems in hypersaline Lake Retba, Senegal. Environ Microbiol. 2011;13(8):1956-72. Epub 2010/08/27. doi: 10.1111/j.1462-2920.2010.02323.x. PubMed PMID: 20738373;
14. Oren A, Bratbak G, Heldal M. Occurrence of virus-like particles in the Dead Sea. Extremophiles : life under extreme conditions. 1997;1(3):143-9. Epub 1997/08/01. doi: 10.1007/s007920050027. PubMed PMID: 9680320;
15. Jumper J, Evans R, Pritzel A, Green T, Figurnov M, Ronneberger O, et al. Highly accurate protein structure prediction with AlphaFold. Nature. 2021;596(7873):583-9. Epub 2021/07/16. doi: 10.1038/s41586-021-03819-2. PubMed PMID: 34265844; PMC8371605.
16. Baek M, DiMaio F, Anishchenko I, Dauparas J, Ovchinnikov S, Lee GR, et al. Accurate prediction of protein structures and interactions using a three-track neural network. Science (New York, NY). 2021;373(6557):871-6. Epub 2021/07/21. doi: 10.1126/science.abj8754. PubMed PMID: 34282049;
17. Oh B, Moyer CL, Hendrix RW, Duda RL. The delta domain of the HK97 major capsid protein is essential for assembly. Virology. 2014;456-457:171-8. Epub 2014/06/04. doi: 10.1016/j.virol.2014.03.022. PubMed PMID: 24889236; PMC4044616.
