# Supplementary material for: Diversity, taxonomy, and evolution of archaeal viruses of the class Caudoviricetes
Source: PLoS Biol. 2021 Nov 9;19(11):e3001442. doi: 10.1371/journal.pbio.3001442 (PMC8651126; doi:10.1371/journal.pbio.3001442)
Supplement: S1 Fig — Comparisons of genomes of (A) representative viruses from the 4 genera in Hafunaviridae, (B) viruses from the 2 genera in Druskaviridae and viruses from the 2 genera in Saparoviridae, (C) HATV-2 from Soleiviridae and HGTV-1 from Halomagnusviridae (singletons), (D) the 3 viruses from Haloferuviridae (3 genera), (E) HATV-3 from Pyrstoviridae, HFTV1 from Haloferuviridae, HSTV-1 from Shortaselviridae and HRTV-28 from Suolaviridae (all singletons), (F) the 2 members from Graaviviridae, (G) the 3 viruses from Vertoviridae (2 genera), (H) psiM2 representing Leisingerviridae and Drs3 from Anaerodiviridae. Putative protein functions are indicated above or below the corresponding ORFs. Genes encoding virus morphogenesis–related proteins are colored in green, whereas replication-related genes are colored in red. Homologous genes shared between viruses are connected by shadings of different degrees of gray based on the amino acid sequence identity. See S1 Table for more information on families and genera. arTV, archaeal tailed virus. (PDF) [file pbio.3001442.s012.pdf]

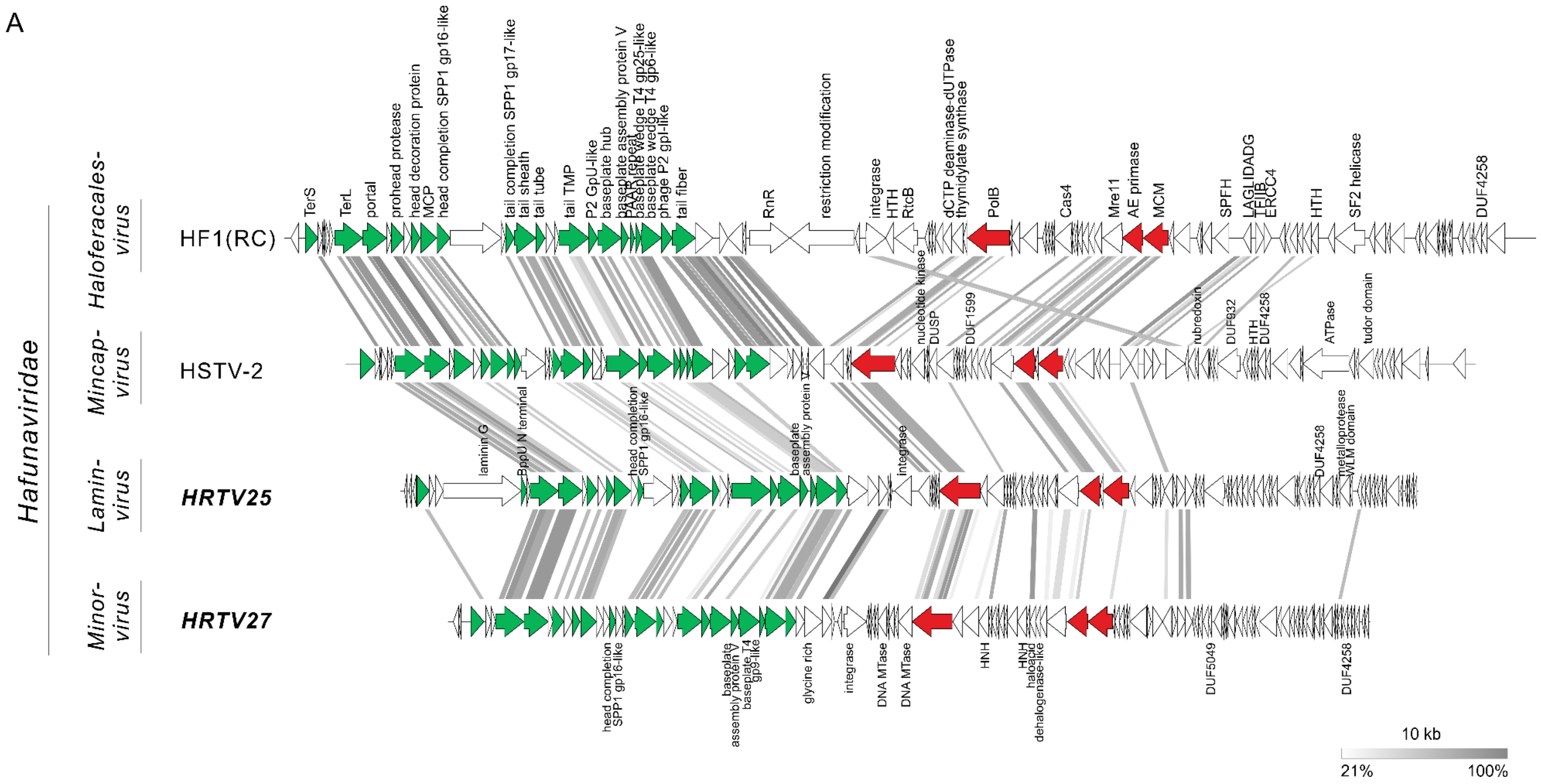
A
Hafunaviridae
Haloferacales-virus
Mincap-virus
Lamin-virus
Minor-virus
HF1(RC)
HSTV-2
HRTV25
HRTV27
TerS
TerL
portal
prohead protease
head decoration protein
MCP
head completion SPP1 gp16-like
tail completion SPP1 gp17-like
tail sheath
tail tube
tail TMP
P2 GpU-like
baseplate hub
baseplate assembly protein V
PAAR repeat
baseplate wedge T4 gp25-like
baseplate wedge T4 gp6-like
phage P2 gpI-like
tail fiber
RnR
restriction modification
integrase
HTH
RtcB
dCTP deaminase-dUTPase
thymidylate synthase
PolB
Cas4
Mre11
AE primase
MCM
SPFH
LAGLIDADG
TFIIB
ERCC4
HTH
SF2 helicase
DUF4258
nucleotide kinase
DUSP
DUF1599
rubredoxin
DUF632
HTH
DUF4258
ATPase
tudor domain
laminin G
BppU N terminal
head completion SPP1 gp16-like
baseplate assembly protein V
integrase
DUF4258
metalloprotease
WLM domain
head completion SPP1 gp16-like
baseplate assembly protein V
baseplate T4 gp9-like
glycine rich
integrase
DNA MTase
DNA MTase
HNH
HNH
haloacid dehalogenase-like
DUF5049
DUF4258
10 kb
21%
100%

B

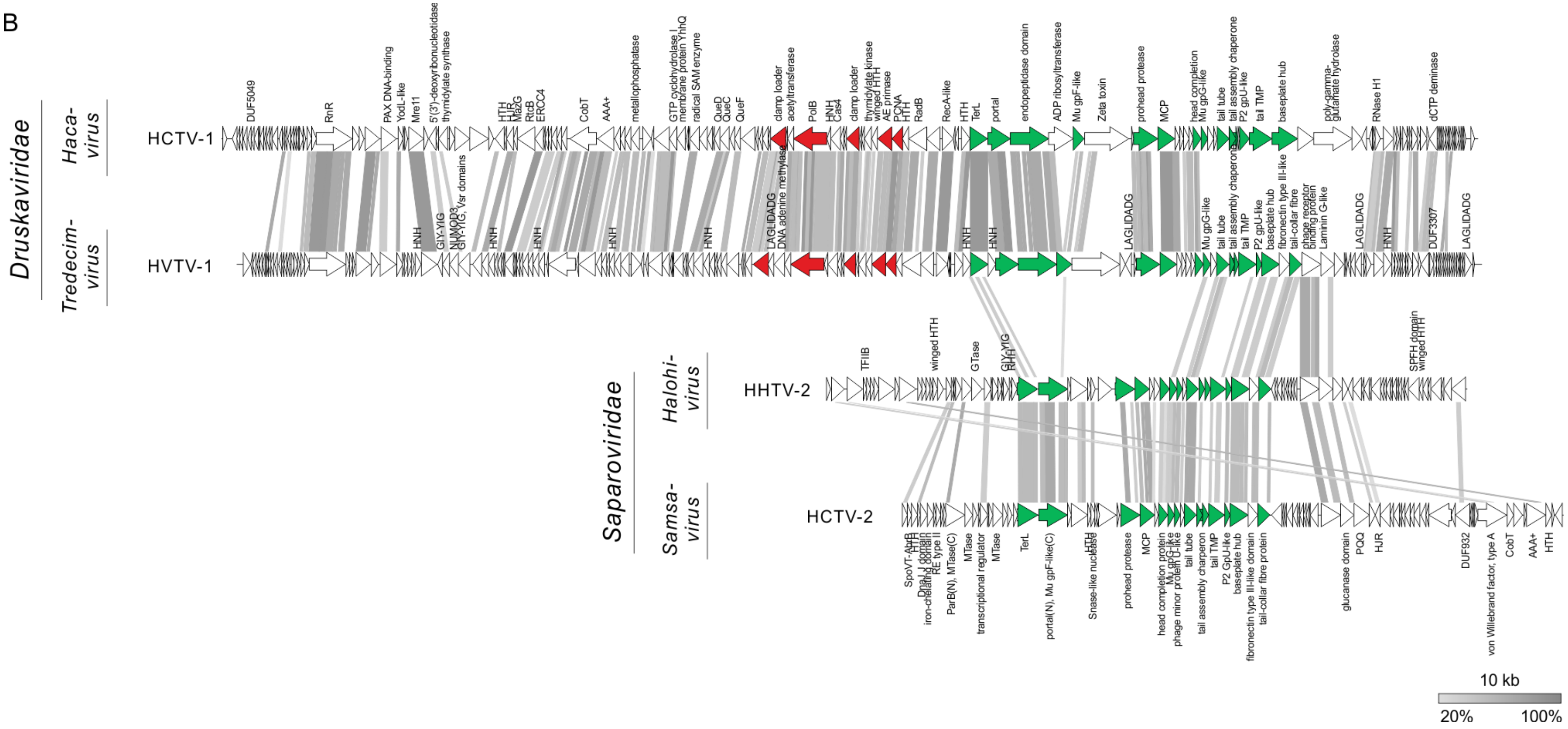

Druskaviridae
Haca-virus
Tredecim-virus
HCTV-1
HVTV-1
Saparoviridae
Halohi-virus
Samsa-virus
HHTV-2
HCTV-2
10 kb
20%
100%

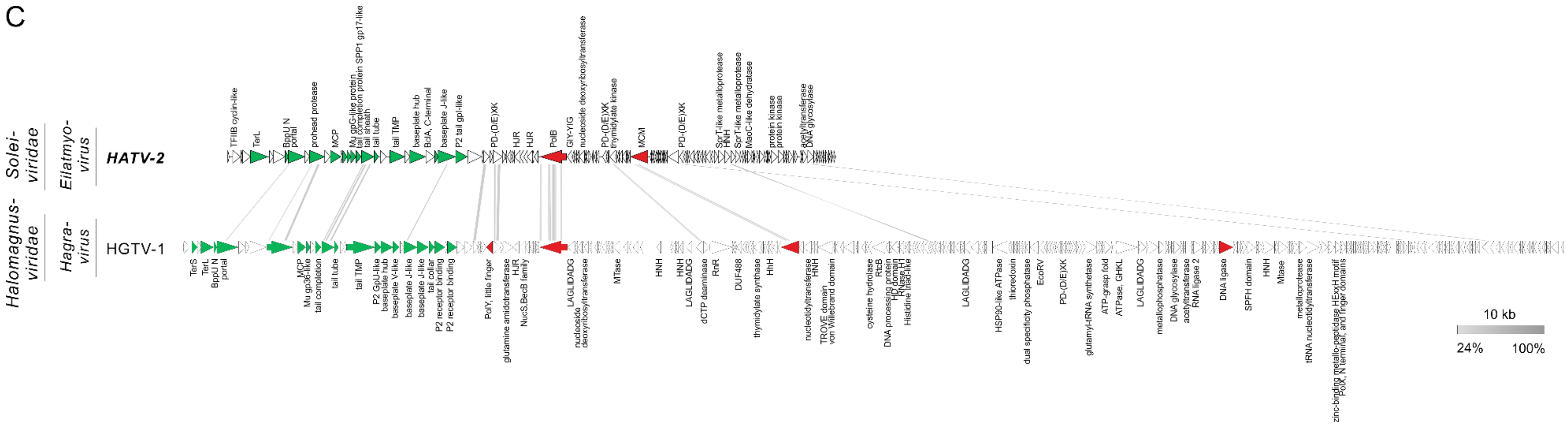
C
Solei-viridae
Eilatmyo-virus
HATV-2
Halomagnus-viridae
Hagra-virus
HGTV-1
TFIIB cyclin-like
TerL
BppU N
portal
prohead protease
MCP
Mu gpG-like protein
tail completion protein SPP1 gp17-like
tail sheath
tail tube
tail TMP
baseplate hub
BclA, C-terminal
baseplate J-like
P2 tail gpI-like
PD-(D/E)XK
HJR
HJR
PolB
GIY-YIG
nucleoside deoxyribosyltransferase
PD-(D/E)XK
thymidylate kinase
MCM
PD-(D/E)XK
SprT-like metalloprotease
HNH
SprT-like metalloprotease
MaoC-like dehydratase
protein kinase
protein kinase
acetyltransferase
DNA glycosylase
TerS
TerL
BppU N
portal
MCP
Mu gp36-like
tail completion
tail tube
tail TMP
P2 GpU-like
baseplate hub
baseplate V-like
baseplate J-like
baseplate J-like
tail collar
P2 receptor binding
P2 receptor binding
PolY, little finger
glutamine amidotransferase
HJR
NucS,BecB family
LAGLIDADG
nucleoside deoxyribosyltransferase
MTase
HNH
HNH
LAGLIDADG
dCTP deaminase
RnR
DUF488
thymidylate synthase
HhH
nucleotidyltransferase
HNH
TROVE domain
von Willebrand domain
cysteine hydrolase
RtcB
DNA processing protein
HD domain
RNase H
Histidine triad-like
LAGLIDADG
HSP90-like ATPase
thioredoxin
dual specificity phosphatase
EcoRV
PD-(D/E)XK
glutamyl-tRNA synthetase
ATP-grasp fold
ATPase, GHKL
LAGLIDADG
metallophosphatase
DNA glycosylase
acetyltransferase
RNA ligase 2
DNA ligase
SPFH domain
HNH
Mtase
metalloprotease
tRNA nucleotidyltransferase
zinc-binding metallo-peptidase HExxH motif
PolX, N terminal, and finger domains
10 kb
24%
100%

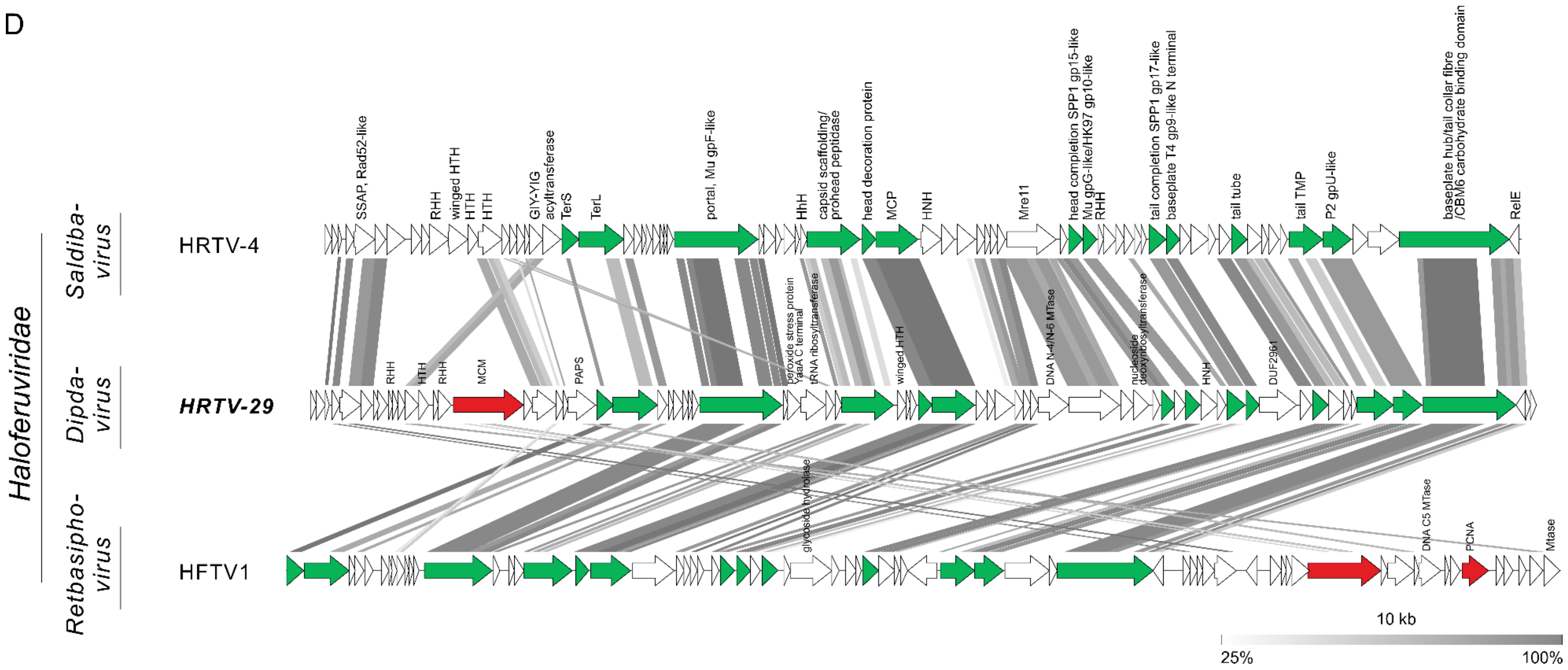
D
Haloferuviridae
Saldibavirus
Dipdavirus
Retbasiphovirus
HRTV-4
HRTV-29
HFTV1
SSAP, Rad52-like
RHH
winged HTH
HTH
HTH
GIY-YIG
acyltransferase
TerS
TerL
portal, Mu gpF-like
HhH
capsid scaffolding/
prohead peptidase
head decoration protein
MCP
HNH
Mre11
head completion SPP1 gp15-like
Mu gpG-like/HK97 gp10-like
RHH
tail completion SPP1 gp17-like
baseplate T4 gp9-like N terminal
tail tube
tail TMP
P2 gpU-like
baseplate hub/tail collar fibre
/CBM6 carbohydrate binding domain
RelE
RHH
HTH
RHH
MCM
PAPS
peroxide stress protein
YaaA C terminal
tRNA ribosyltransferase
winged HTH
DNA N-4/N-6 MTase
nucleoside
deoxyribosyltransferase
HNH
DUF2961
glycoside hydrolase
DNA C5 MTase
PCNA
Mtase
10 kb
25%
100%

E

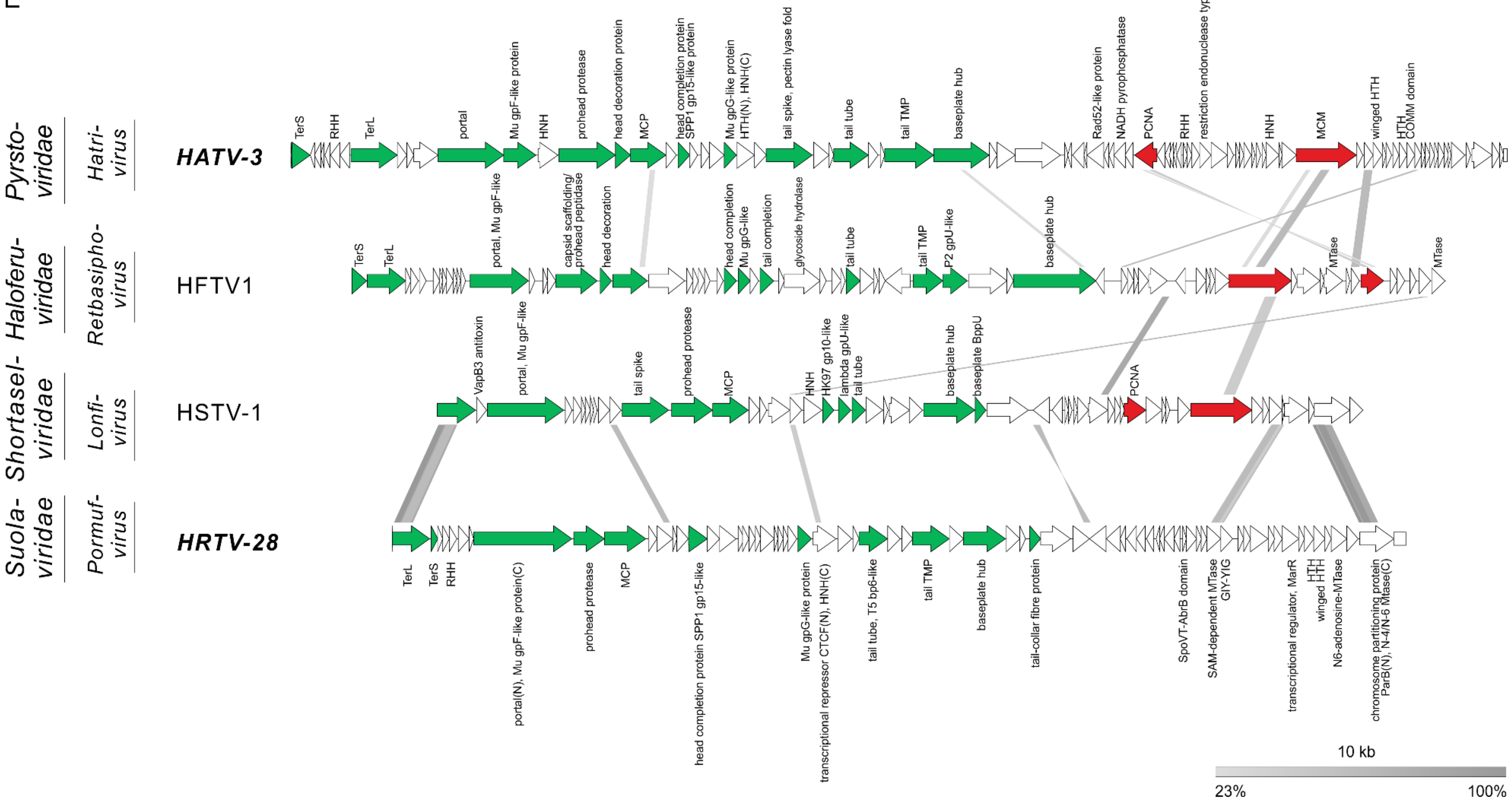

Pyrsto-viridae
Hatri-virus
HATV-3
TerS
RHH
TerL
portal
Mu gpF-like protein
HNH
prohead protease
head decoration protein
MCP
head completion protein
SPP1 gp15-like protein
Mu gpG-like protein
HTH(N), HNH(C)
tail spike, pectin lyase fold
tail tube
tail TMP
baseplate hub
Rad52-like protein
NADH pyrophosphatase
PCNA
RHH
restriction endonuclease type II
HNH
MCM
winged HTH
HTH
COMM domain
Haloferu-viridae
Retbasipho-virus
HFTV1
TerS
TerL
portal, Mu gpF-like
capsid scaffolding/
prohead peptidase
head decoration
head completion
Mu gpG-like
tail completion
glycoside hydrolase
tail tube
tail TMP
P2 gpU-like
baseplate hub
MTase
MTase
Shortasel-viridae
Lonfi-virus
HSTV-1
VapB3 antitoxin
portal, Mu gpF-like
tail spike
prohead protease
MCP
HNH
HK97 gp10-like
lambda gpU-like
tail tube
baseplate hub
baseplate BppU
PCNA
Suola-viridae
Pormuf-virus
HRTV-28
TerL
TerS
RHH
portal(N), Mu gpF-like protein(C)
prohead protease
MCP
head completion protein SPP1 gp15-like
Mu gpG-like protein
transcriptional repressor CTCF(N), HNH(C)
tail tube, T5 bp6-like
tail TMP
baseplate hub
tail-collar fibre protein
SpoVT-AbrB domain
SAM-dependent MTase
GIY-YIG
transcriptional regulator, MarR
HTH
winged HTH
N6-adenosine-MTase
chromosome partitioning protein
ParB(N), N-4/N-6 Mtase(C)
10 kb
23%
100%

F

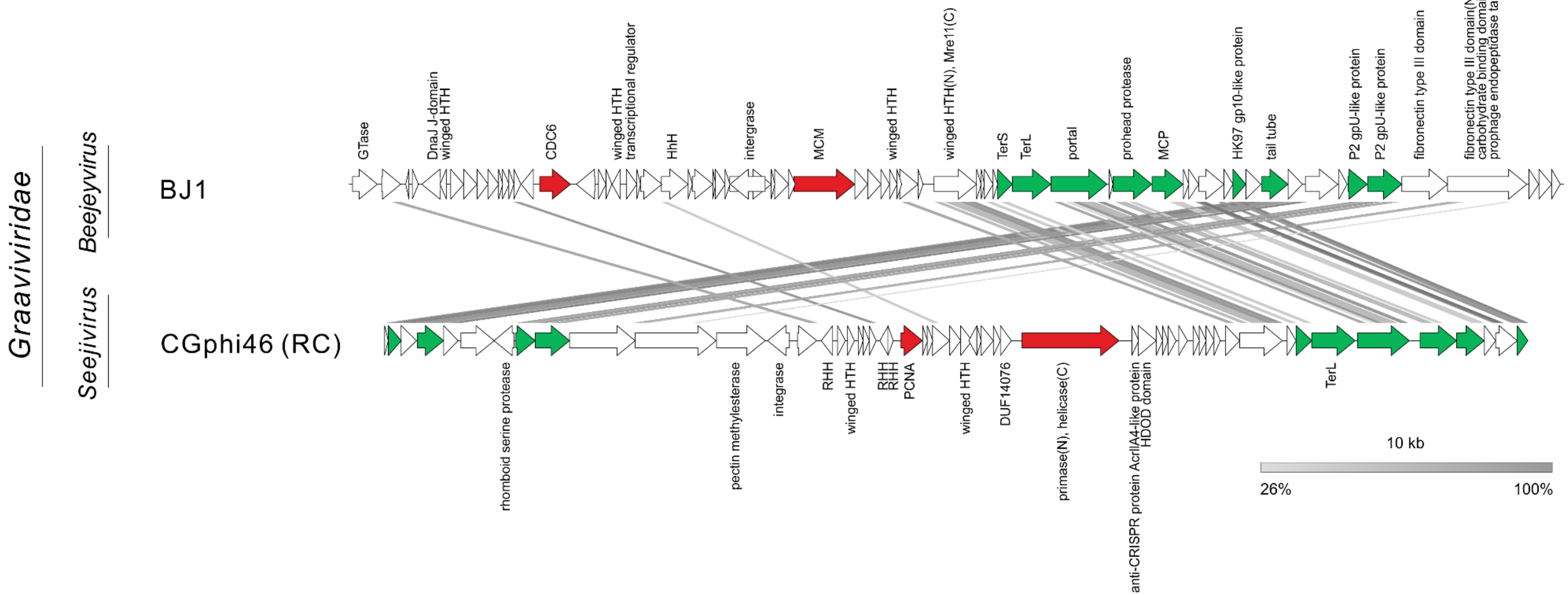

Graaviviridae
Beejeyvirus
Seejivirus
BJ1
CGphi46 (RC)
GTase
DnaJ J-domain
winged HTH
CDC6
winged HTH
transcriptional regulator
HhH
intergrase
MCM
winged HTH
winged HTH(N), Mre11(C)
TerS
TerL
portal
prohead protease
MCP
HK97 gp10-like protein
tail tube
P2 gpU-like protein
P2 gpU-like protein
fibronectin type III domain
fibronectin type III domain(N),
carbohydrate binding domain(M),
prophage endopeptidase tail(C)
rhomboid serine protease
pectin methylesterase
integrase
RHH
winged HTH
RHH
RHH
PCNA
winged HTH
DUF14076
primase(N), helicase(C)
anti-CRISPR protein AcrIIA4-like protein
HDOD domain
TerL
10 kb
26%
100%

G

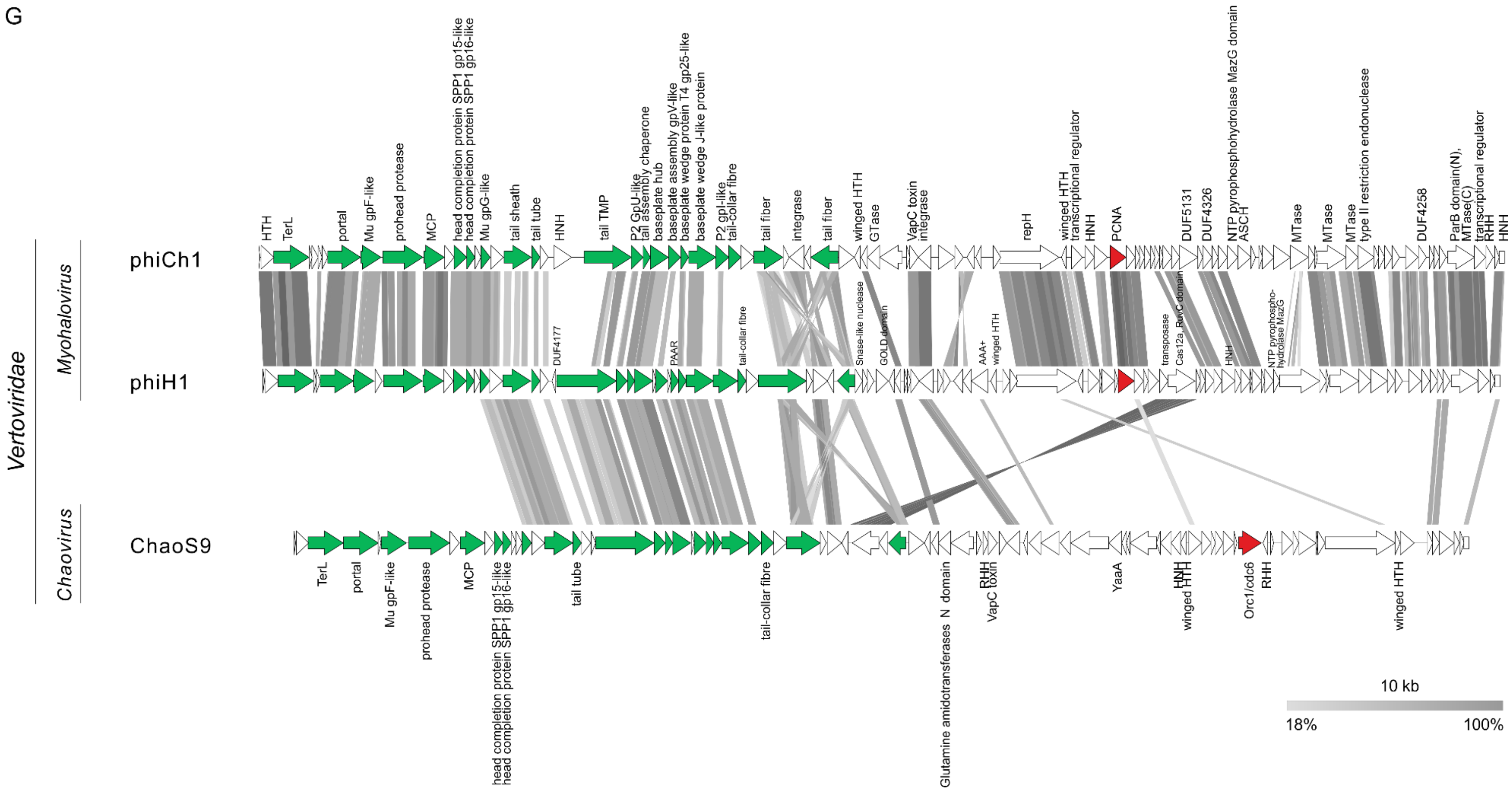

Vertoviridae
Myohalovirus
Chaovirus
phiCh1
phiH1
ChaoS9
HTH
TerL
portal
Mu gpF-like
prohead protease
MCP
head completion protein SPP1 gp15-like
head completion protein SPP1 gp16-like
Mu gpG-like
tail sheath
tail tube
HNH
tail TMP
P2 GpU-like
tail assembly chaperone
baseplate hub
baseplate assembly gpV-like
baseplate wedge protein T4 gp25-like
baseplate wedge J-like protein
P2 gpI-like
tail-collar fibre
tail fiber
integrase
tail fiber
winged HTH
GTase
VapC toxin
integrase
repH
winged HTH
transcriptional regulator
HNH
PCNA
DUF5131
DUF4326
NTP pyrophosphohydrolase MazG domain
ASCH
MTase
MTase
MTase
type II restriction endonuclease
DUF4258
ParB domain(N),
MTase(C)
transcriptional regulator
RHH
HNH
DUF4177
PAAR
tail-collar fibre
Snase-like nuclease
GOLD domain
AAA+
winged HTH
transposase
Cas12a_RuvC domain
HNH
NTP pyrophospho-
hydrolase MazG
TerL
portal
Mu gpF-like
prohead protease
MCP
head completion protein SPP1 gp15-like
head completion protein SPP1 gp16-like
tail tube
tail-collar fibre
Glutamine amidotransferases N domain
RHH
VapC toxin
YaaA
HNH
winged HTH
Orc1/cdc6
RHH
winged HTH
10 kb
18%
100%

H

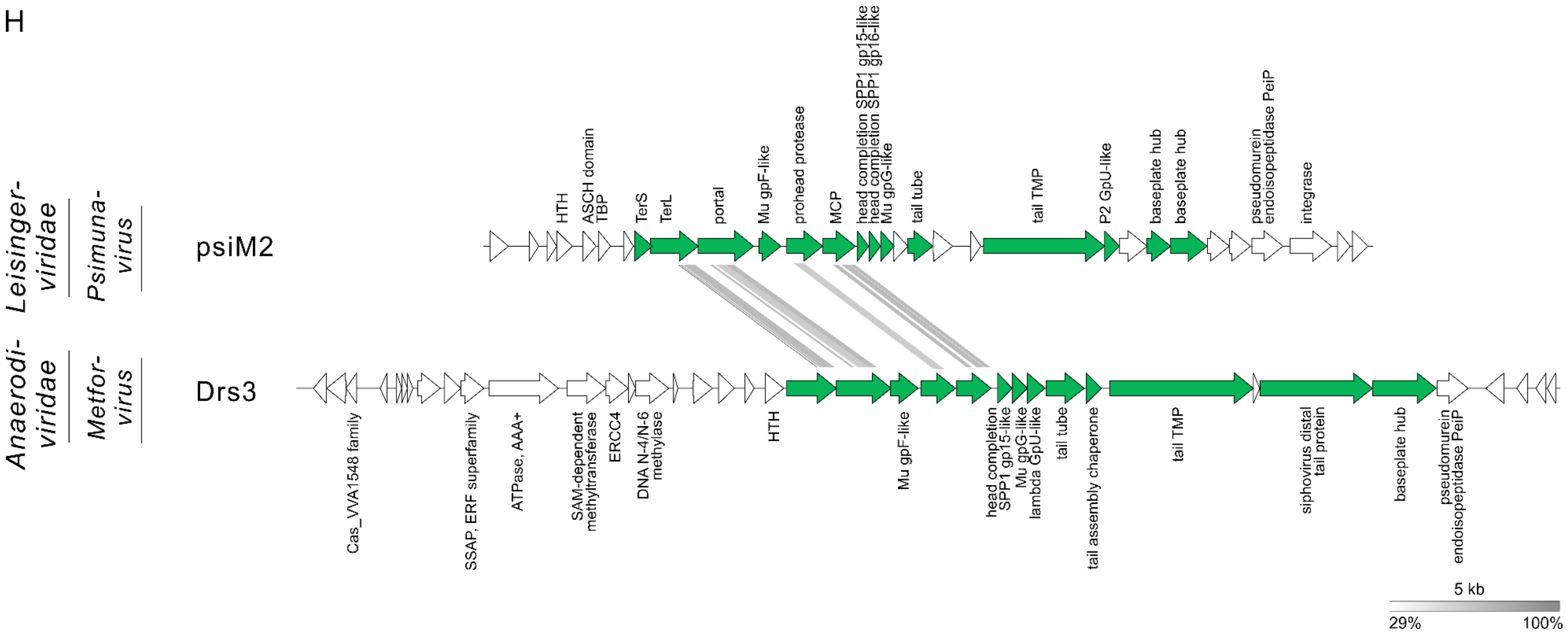

S1 Fig. Genome alignment of archaeal tailed viruses. Comparisons of genomes of (A) representative viruses from the four genera in *Hafunaviridae*, (B) viruses from the two genera in *Druskaviridae* and viruses from the two genera in *Saparoviridae*, (C) HATV-2 from *Soleiviridae* and HGTV-1 from *Halomagnusviridae* (singletons), (D) the three viruses from *Haloferuviridae* (three genera) (E) HATV-3 from *Pyrstoviridae*, HFTV1 from *Haloferuviridae*, HSTV-1 from *Shortaselviridae* and HRTV-28 from *Suolaviridae* (all singletons), (F) the two members from *Graaviviridae*, (G) the three viruses from *Vertoviridae* (two genera), (H) psiM2 representing *Leisingerviridae* and Drs3 from *Anaerodiviridae*. Putative protein functions are indicated above or below the corresponding ORFs. Genes encoding virus morphogenesis related proteins are colored in green, whereas replication related genes are colored in red. Homologous genes shared between viruses are connected by shadings of different degrees of grey based on the amino acid sequence identity. See S1 Table for more information on families and genera.
