# Supplementary material for: Diversity, taxonomy, and evolution of archaeal viruses of the class Caudoviricetes
Source: PLoS Biol. 2021 Nov 9;19(11):e3001442. doi: 10.1371/journal.pbio.3001442 (PMC8651126; doi:10.1371/journal.pbio.3001442)
Supplement: S2 Fig — The percentage of genes of a representative virus from each genus that shared with members of the proposed genus (G) and family (F), as well as arTVs from other families (A) are shown. Each box represents the middle 50th percentile of the data set and is derived using the lower and upper quartile values. The median value is displayed by a horizontal line. Whiskers represent the maximum and minimum values with the range of 1.5 IQR. Each virus is represented by dots. Proteins with over 30% amino acid sequence identity and E-value < 1 × 10^−25^ in our arTV database are counted as homologous proteins. Data underlying this figure can be found in S2 Data. arTV, archaeal tailed virus. (PDF) [file pbio.3001442.s013.pdf]

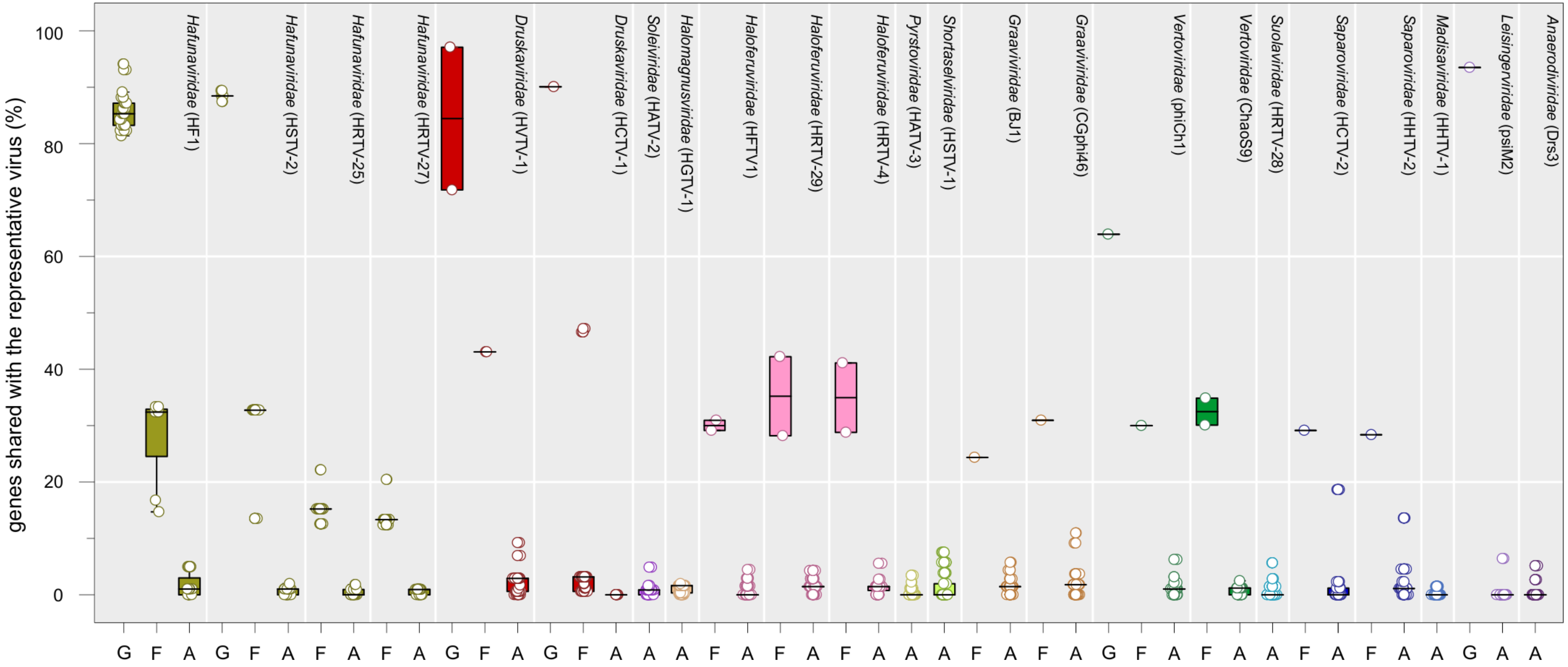

S2 Fig. The box plot shows the percentage of genes shared by arTVs. The percentage of genes of a representative virus from each genus that shared with members of the proposed genus (G) and family (F), as well as arTVs from other families (A) are shown. Each box represents the middle 50th percentile of the data set and is derived using the lower and upper quartile values. The median value is displayed by a horizontal line. Whiskers represent the maximum and minimum values with the range of 1.5 IQR. Each virus is represented by dots. Proteins with over 30% amino acid sequence identity and E-value < 1×10^{-25} in our arTV database are counted as homologous proteins. Data underlying this figure can be found in S2 Data.
