# Supplementary material for: Diversity, taxonomy, and evolution of archaeal viruses of the class Caudoviricetes
Source: PLoS Biol. 2021 Nov 9;19(11):e3001442. doi: 10.1371/journal.pbio.3001442 (PMC8651126; doi:10.1371/journal.pbio.3001442)
Supplement: S3 Fig — The boxed signature motifs are used to classify the MTases. arTV, archaeal tailed virus; MTase, methyltransferase. (PDF) [file pbio.3001442.s014.pdf]

N6-adenine MTase group1

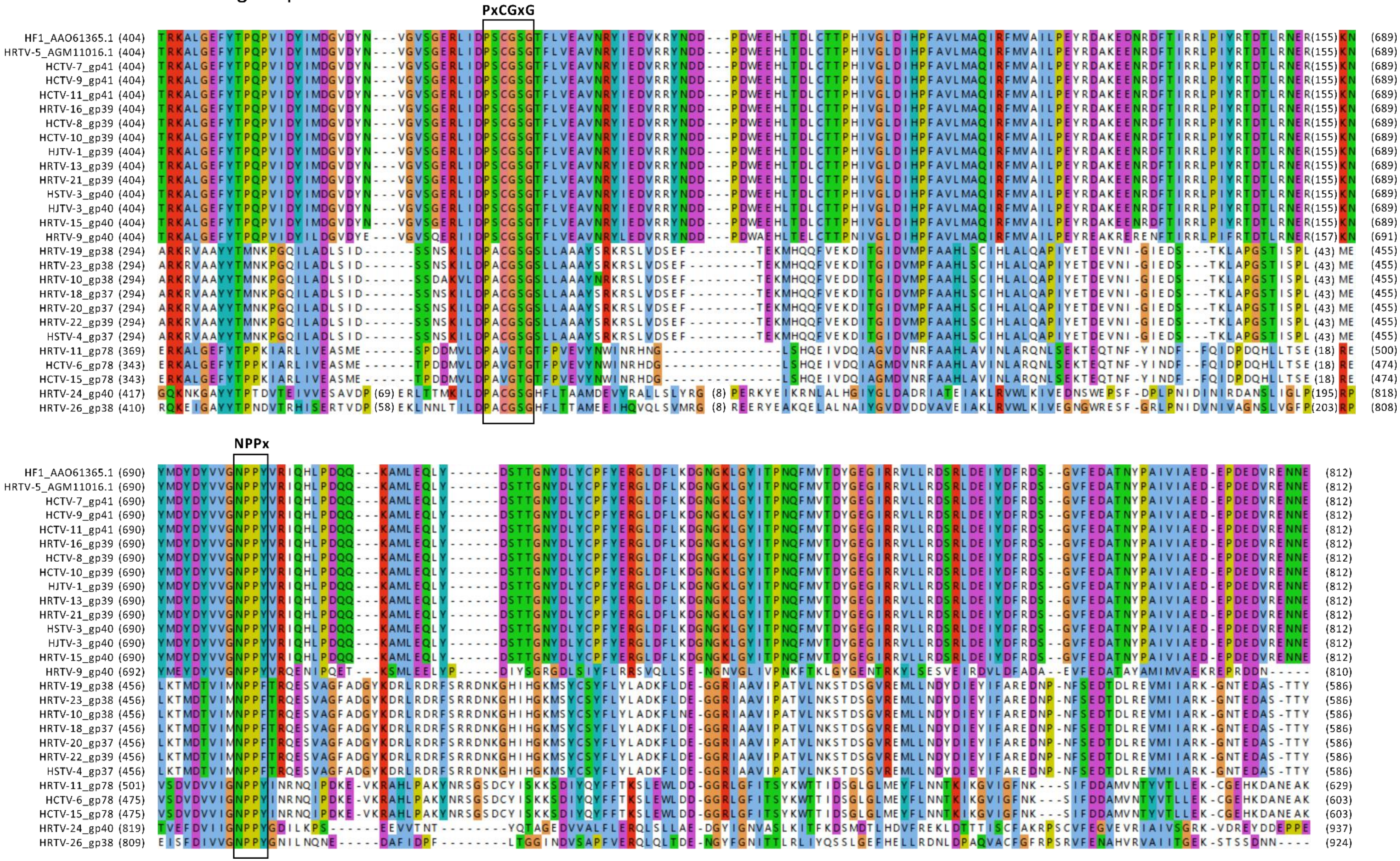

N6-adenine MTase group2 Dam-like

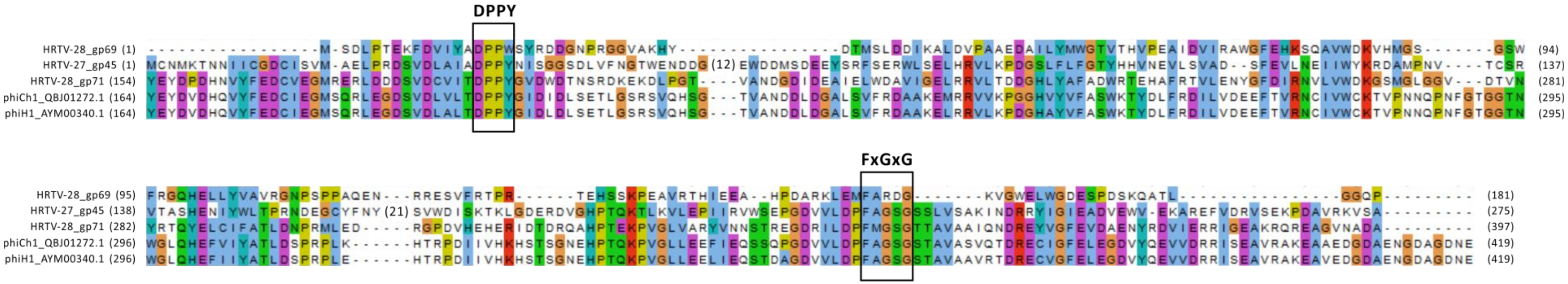

N6-adenine MTase group3

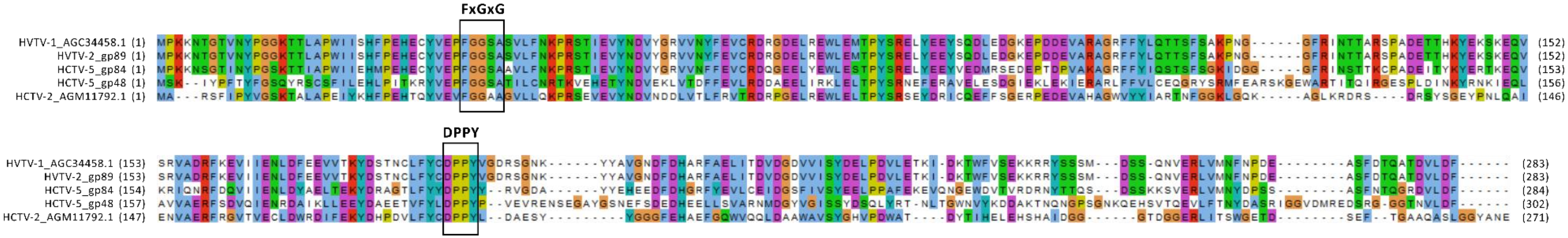

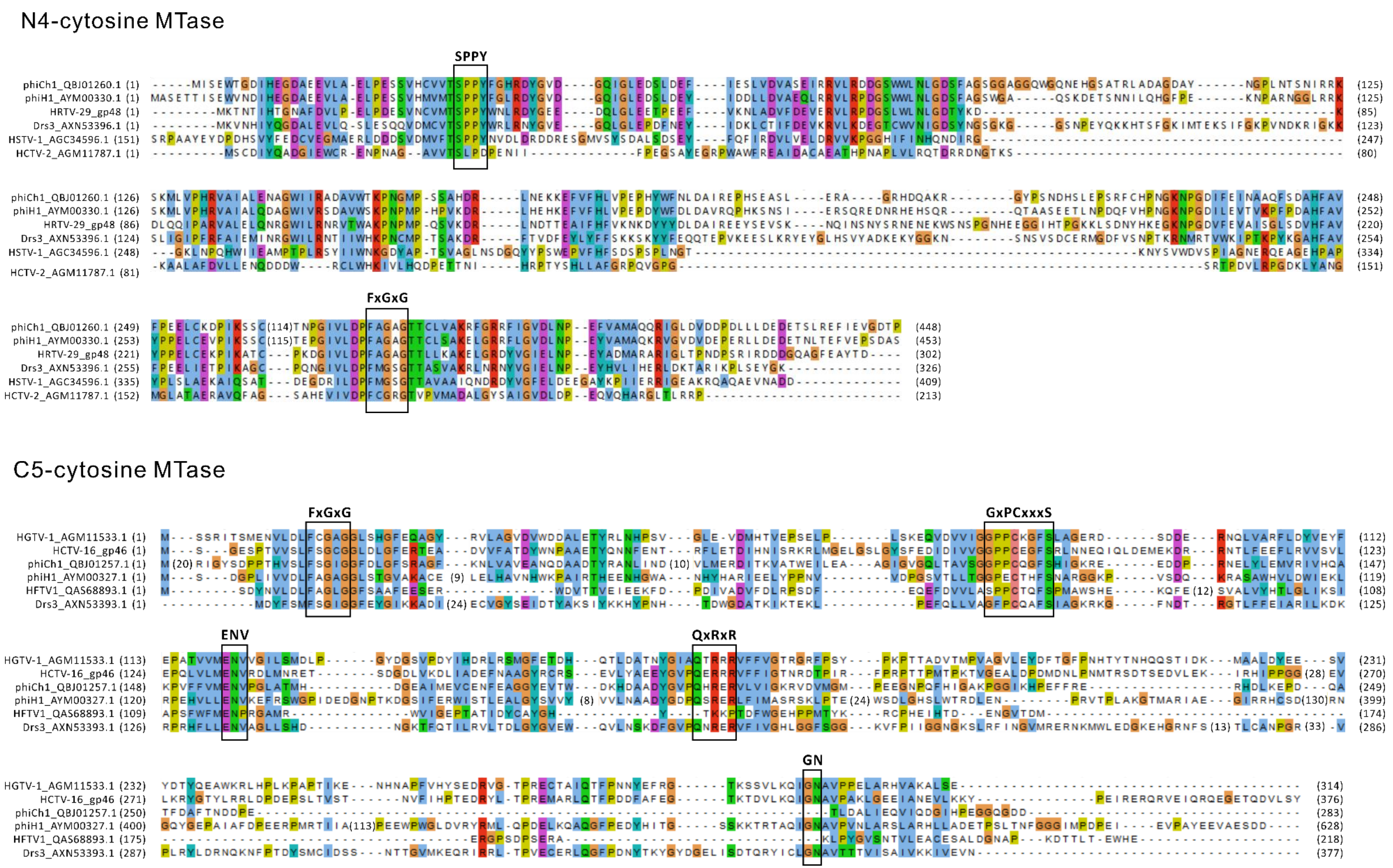

S3 Fig. Multiple sequence alignments of different categories of MTases encoded by arTVs. The boxed signature motifs are used to classify the MTases.
