# Supplementary material for: Diversity, taxonomy, and evolution of archaeal viruses of the class Caudoviricetes
Source: PLoS Biol. 2021 Nov 9;19(11):e3001442. doi: 10.1371/journal.pbio.3001442 (PMC8651126; doi:10.1371/journal.pbio.3001442)
Supplement: S4 Fig — The experimentally verified micrococcal nuclease encoded by Staphylococcus hyicus (AAA26661.1) is used as a sequence reference in the alignment. The empty and filled stars indicate the calcium binding and catalytic sites, respectively. arTV, archaeal tailed virus. (PDF) [file pbio.3001442.s015.pdf]

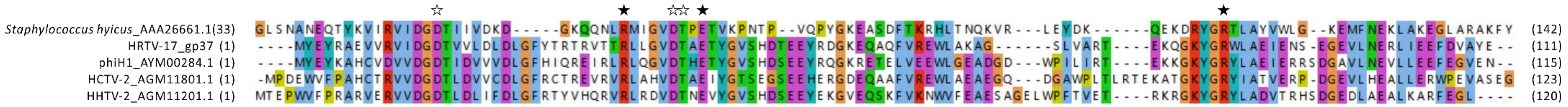

S4 Fig. Multiple sequence alignment of micrococcal nucleases encoded by archaeal tailed viruses. The experimentally verified micrococcal nuclease encoded by *Staphylococcus hyicus* (AAA26661.1) is used as a sequence reference in the alignment. The empty and filled stars indicate the calcium binding and catalytic sites, respectively.
