# Supplementary material for: Diversity, taxonomy, and evolution of archaeal viruses of the class Caudoviricetes
Source: PLoS Biol. 2021 Nov 9;19(11):e3001442. doi: 10.1371/journal.pbio.3001442 (PMC8651126; doi:10.1371/journal.pbio.3001442)
Supplement: S6 Fig — Genes encoding virus morphogenesis proteins, genome replication proteins and integrases are colored in green, red and orange, respectively. For visualization purposes, genomes of some proviruses were circularized and reopened at a different position, matching the start site of the reference arTV genome. Homologous genes shared between (pro)viruses are connected by shadings of different degrees of gray based on the amino acid sequence identity. See S7 Table for complete lists of proviruses of Hafunaviridae, Haloferuviridae, Graaviviridae, Vertoviridae, Leisingerviridae, and Anaerodiviridae. arTV, archaeal tailed virus; att, attachment site. (PDF) [file pbio.3001442.s017.pdf]

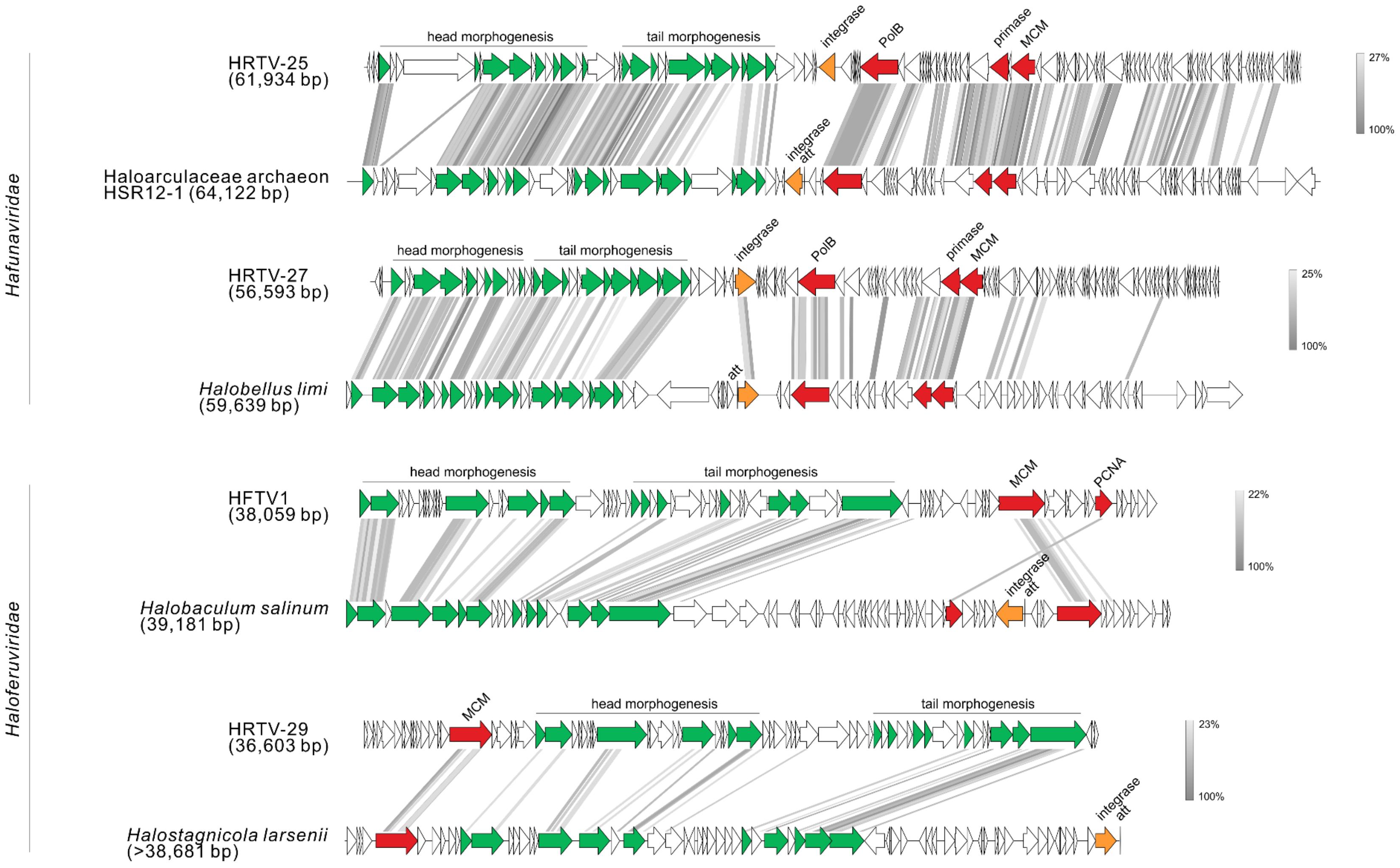

Hafunaviridae
Haloferuviridae
head morphogenesis
tail morphogenesis
integrase
PolB
primase
MCM
HRTV-25
(61,934 bp)
27%
100%
integrase
att
Haloarculaceae archaeon
HSR12-1 (64,122 bp)
HRTV-27
(56,593 bp)
25%
100%
att
Halobellus limi
(59,639 bp)
PCNA
HFTV1
(38,059 bp)
22%
100%
Halobaculum salinum
(39,181 bp)
HRTV-29
(36,603 bp)
23%
100%
Halostagnicola larsenii
(>38,681 bp)

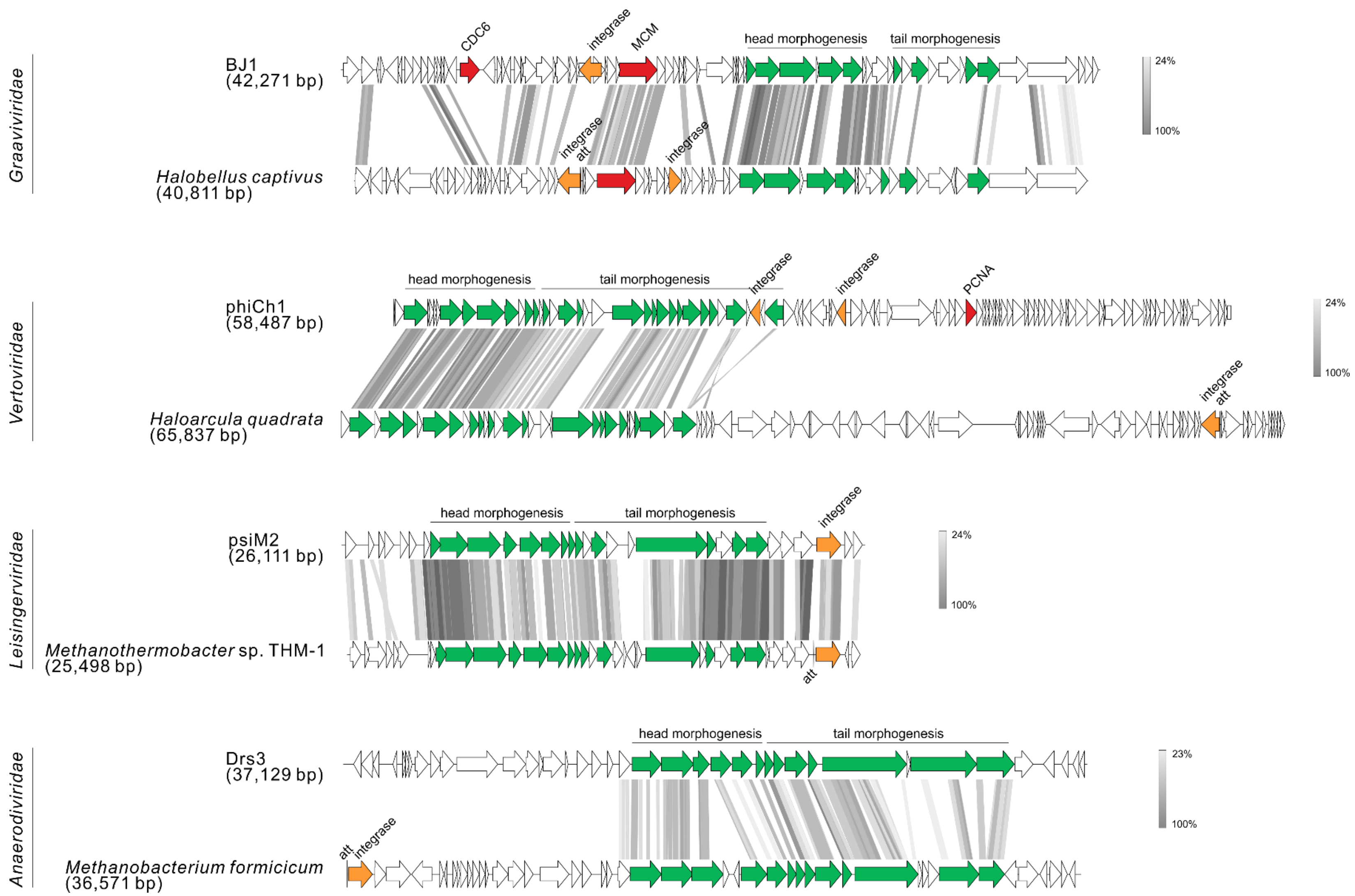

Graaviviridae
BJ1
(42,271 bp)
CDC6
integrase
MCM
head morphogenesis
tail morphogenesis
Halobellus captivus
(40,811 bp)
integrase
att
integrase
24%
100%
Vertoviridae
phiCh1
(58,487 bp)
head morphogenesis
tail morphogenesis
integrase
integrase
PCNA
Haloarcula quadrata
(65,837 bp)
integrase
att
24%
100%
Leisingerviridae
psiM2
(26,111 bp)
head morphogenesis
tail morphogenesis
integrase
Methanothermobacter sp. THM-1
(25,498 bp)
att
24%
100%
Anaerodiviridae
Drs3
(37,129 bp)
head morphogenesis
tail morphogenesis
Methanobacterium formicicum
(36,571 bp)
att
integrase
23%
100%

S6 Fig. Genome comparisons of the representative proviruses with related arTVs from six viral families. Genes encoding virus morphogenesis proteins, genome replication proteins and integrases are colored in green, red and orange, respectively. For visualization purposes, genomes of some proviruses were circularized and reopened at a different position, matching the start site of the reference arTV genome. Homologous genes shared between (pro)viruses are connected by shadings of different degrees of grey based on the amino acid sequence identity. See S7 Table for complete lists of proviruses of *Hafunaviridae*, *Haloferuviridae*, *Graaviviridae*, *Vertoviridae*, *Leisingerviridae* and *Anaerodiviridae*. *att*, attachment site.
