# Supplementary material for: Diversity, taxonomy, and evolution of archaeal viruses of the class Caudoviricetes
Source: PLoS Biol. 2021 Nov 9;19(11):e3001442. doi: 10.1371/journal.pbio.3001442 (PMC8651126; doi:10.1371/journal.pbio.3001442)
Supplement: S7 Fig — Putative protein functions are indicted above the corresponding ORFs. Genes encoding virus morphogenesis related proteins are colored in green, whereas replication-related genes are in red. The putative tail fiber adhesin-encoding gene is shown in yellow. The genes encoding restriction modification enzymes are in blue. Homologous genes shared between viruses are connected by shadings of different degrees of gray based on the amino acid sequence identity. (PDF) [file pbio.3001442.s018.pdf]

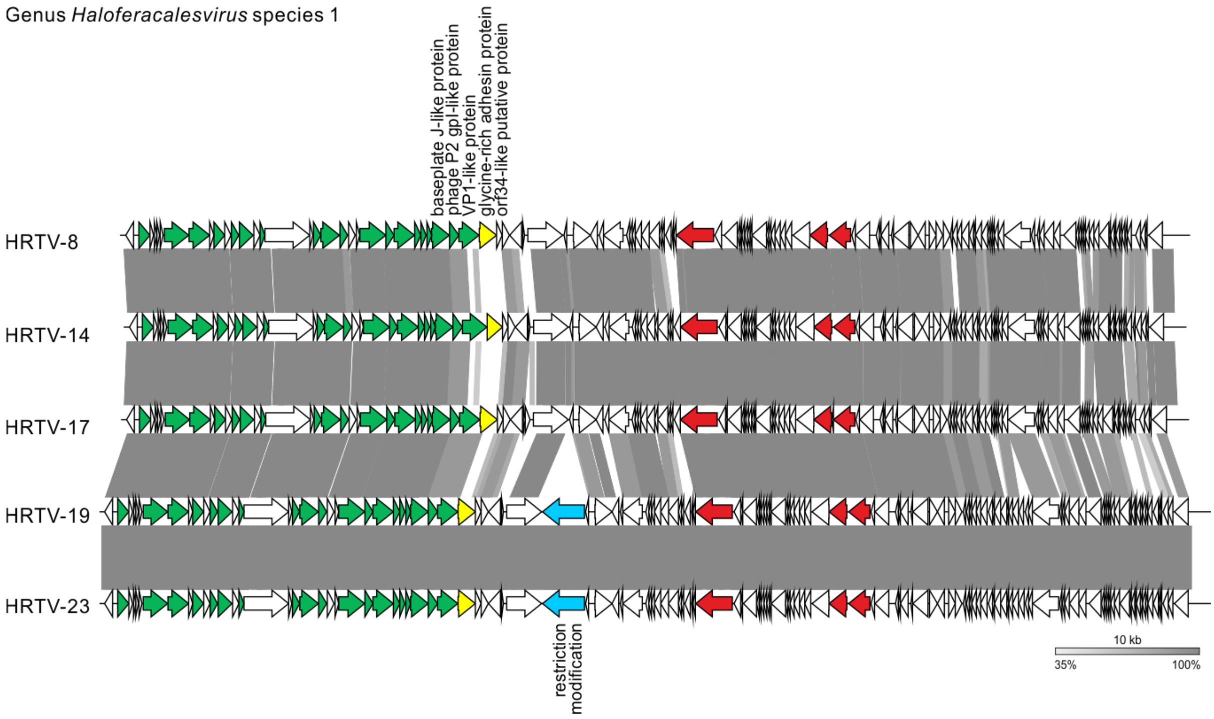
Genus *Haloferacalesvirus* species 1
baseplate J-like protein
phage P2 gpI-like protein
VP1-like protein
glycine-rich adhesin protein
orf34-like putative protein
HRTV-8
HRTV-14
HRTV-17
HRTV-19
HRTV-23
restriction
modification
10 kb
35%
100%

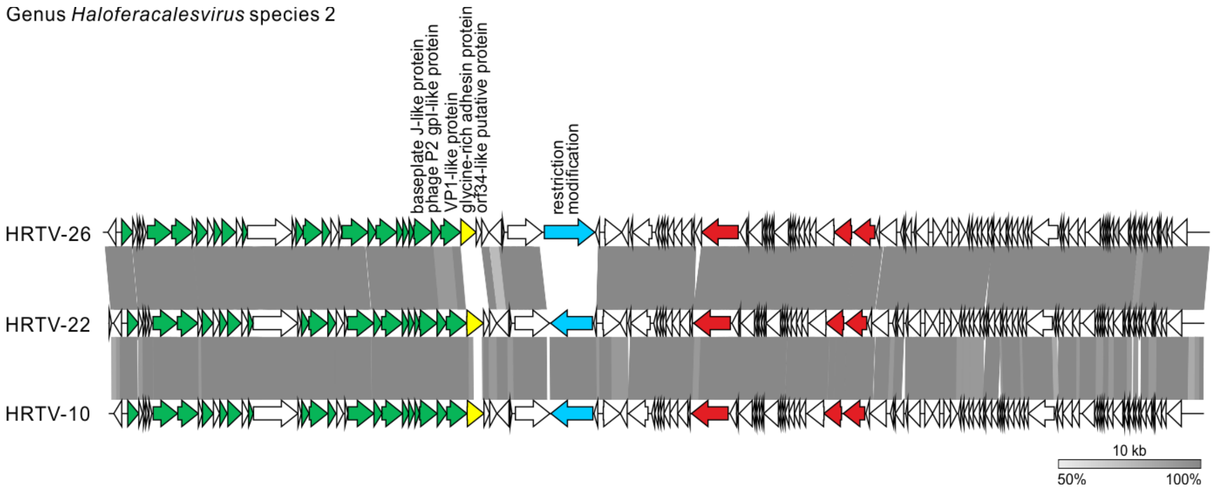
Genus *Haloferacalesvirus* species 2
baseplate J-like protein
phage P2 gpI-like protein
VP1-like protein
glycine-rich adhesin protein
orf34-like putative protein
restriction
modification
HRTV-26
HRTV-22
HRTV-10
10 kb
50%
100%

Genus *Haloferacalesvirus* species 4

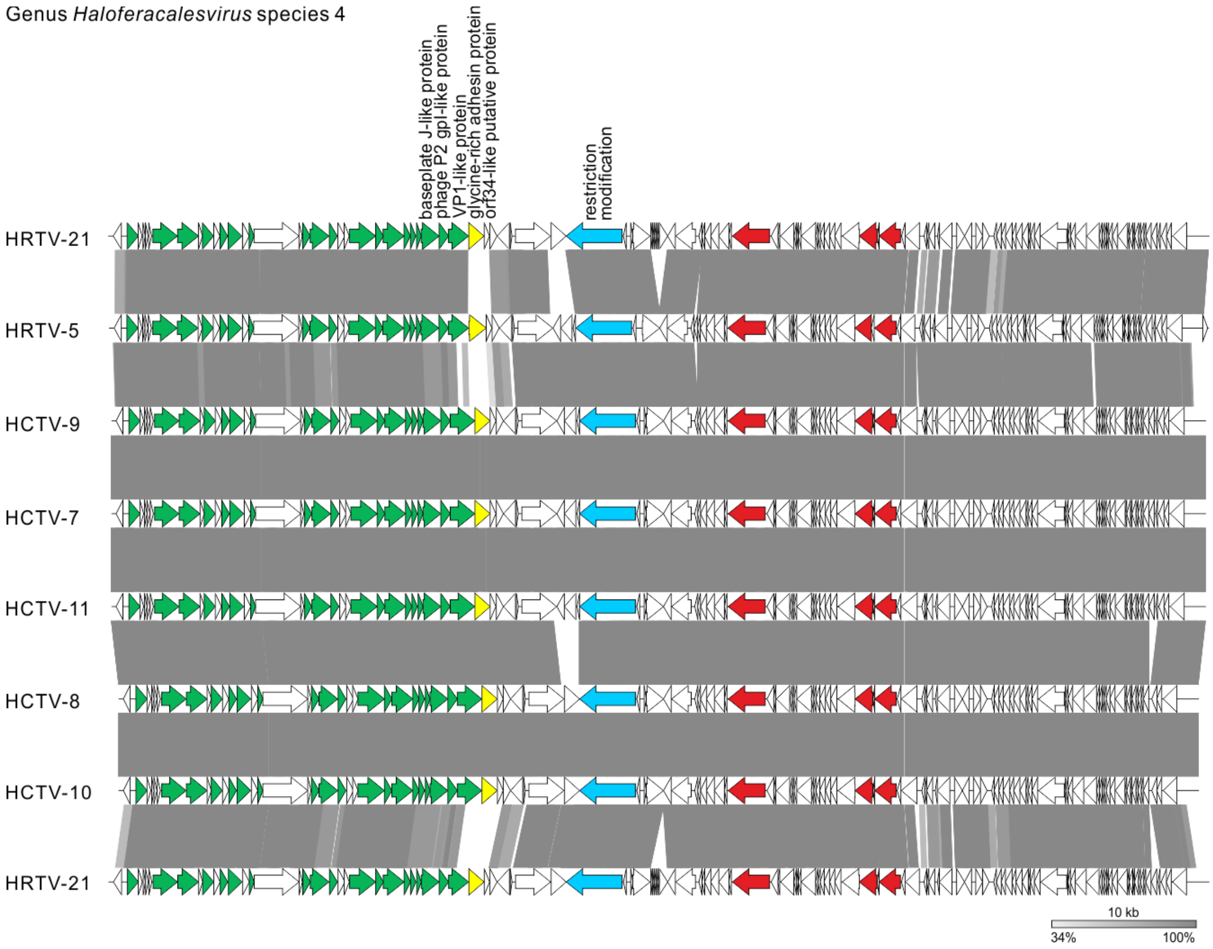

Genus *Haloferacalesvirus* species 5

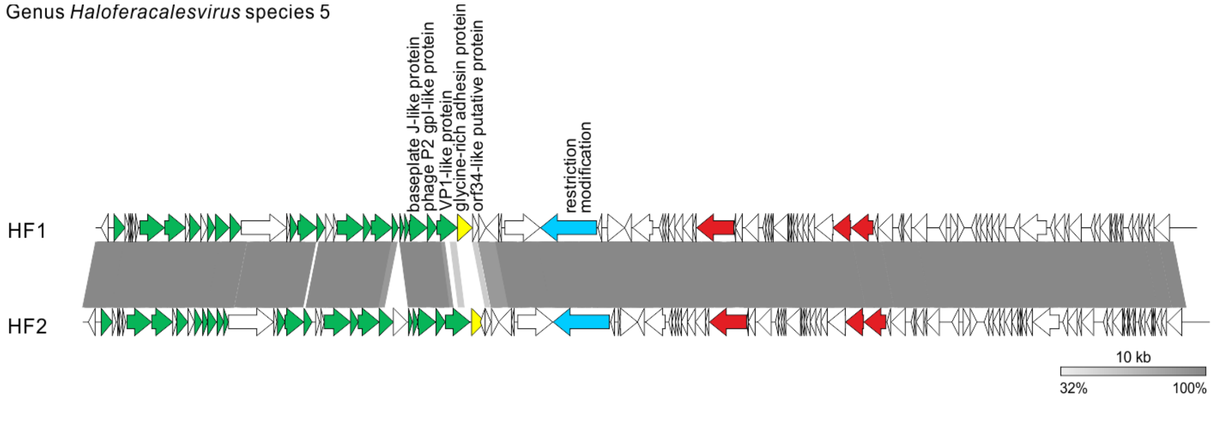

Genus *Haloferacalesvirus* species 6

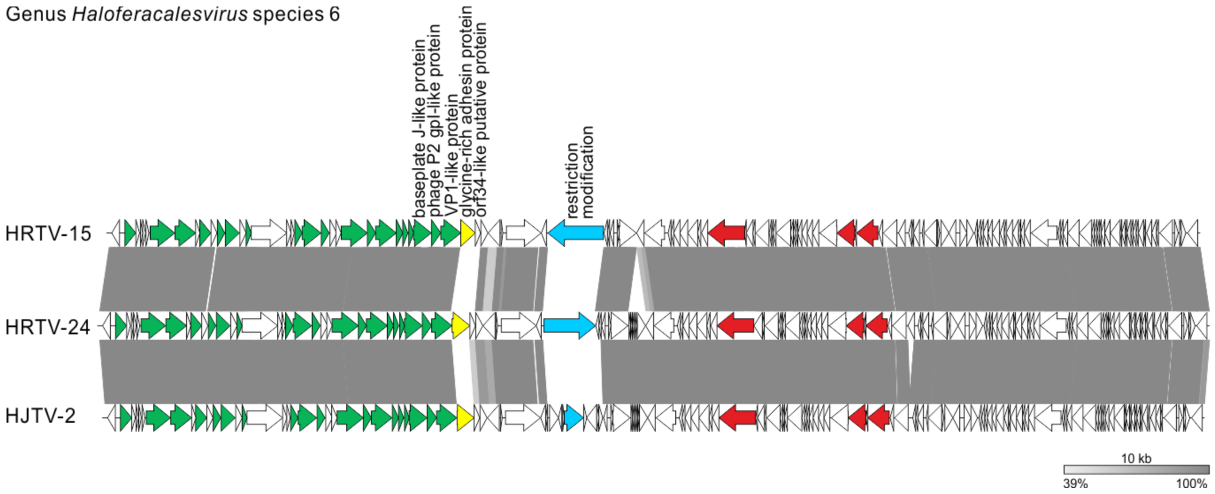

Genus *Mincapvirus* species 1

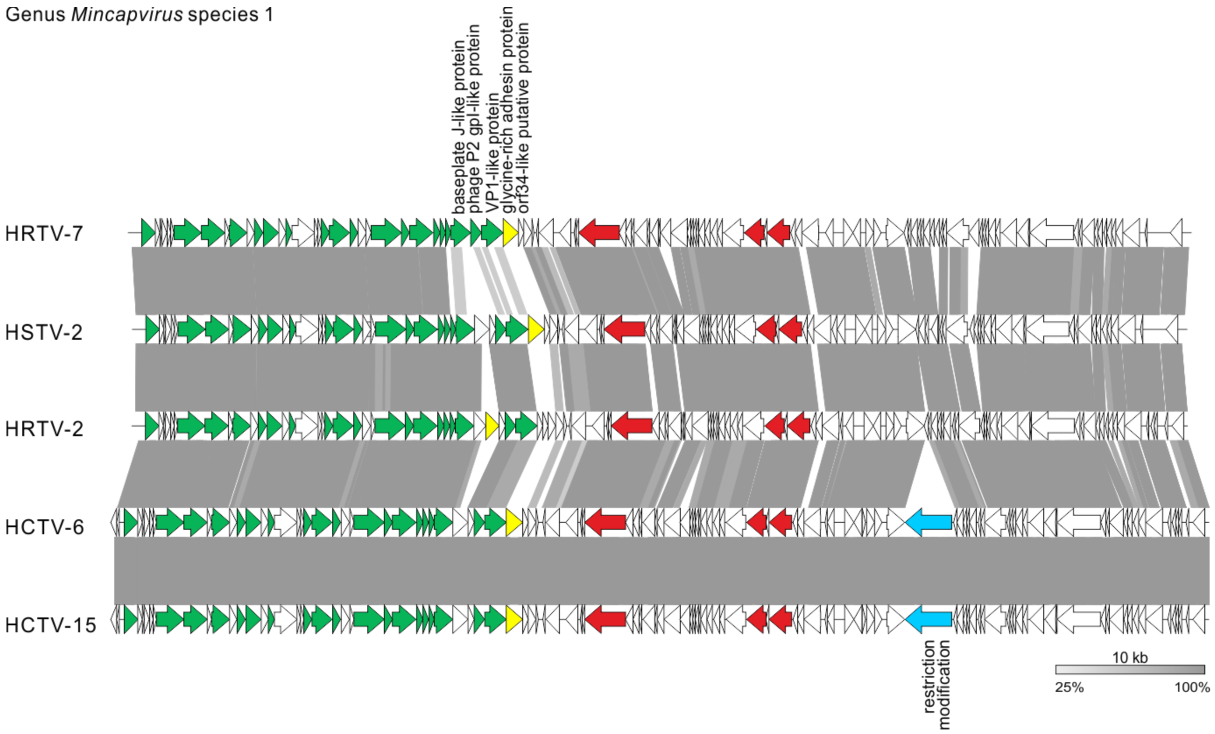

S7 Fig. The pairwise genome comparisons of viruses from the genera *Haloferacalesvirus* and *Mincapvirus* of the family *Hafunaviridae*. Putative protein functions are indicted above the corresponding ORFs. Genes encoding virus morphogenesis related proteins are colored in green, whereas replication related genes are in red. The putative tail fiber adhesin-encoding gene is shown in yellow. The genes encoding restriction modification enzymes are in blue. Homologous genes shared between viruses are connected by shadings of different degrees of grey based on the amino acid sequence identity.
