# Supplementary material for: Diversity, taxonomy, and evolution of archaeal viruses of the class Caudoviricetes
Source: PLoS Biol. 2021 Nov 9;19(11):e3001442. doi: 10.1371/journal.pbio.3001442 (PMC8651126; doi:10.1371/journal.pbio.3001442)
Supplement: S8 Fig — (A) Pairwise sequence alignments of adhesins encoded by viruses from the genera Haloferacalesvirus and Mincapvirus. The HVSs, which are predicted to interact with host receptor, are indicated. (B) Phylogeny of the adhesins. Viruses that were used for EOP assay in this study are indicated in black, otherwise in gray. Nodes with bootstrap support values greater than 90% are indicated with dots. EOP, efficiency of plating; HVS, hypervariable segment. (PDF) [file pbio.3001442.s019.pdf]

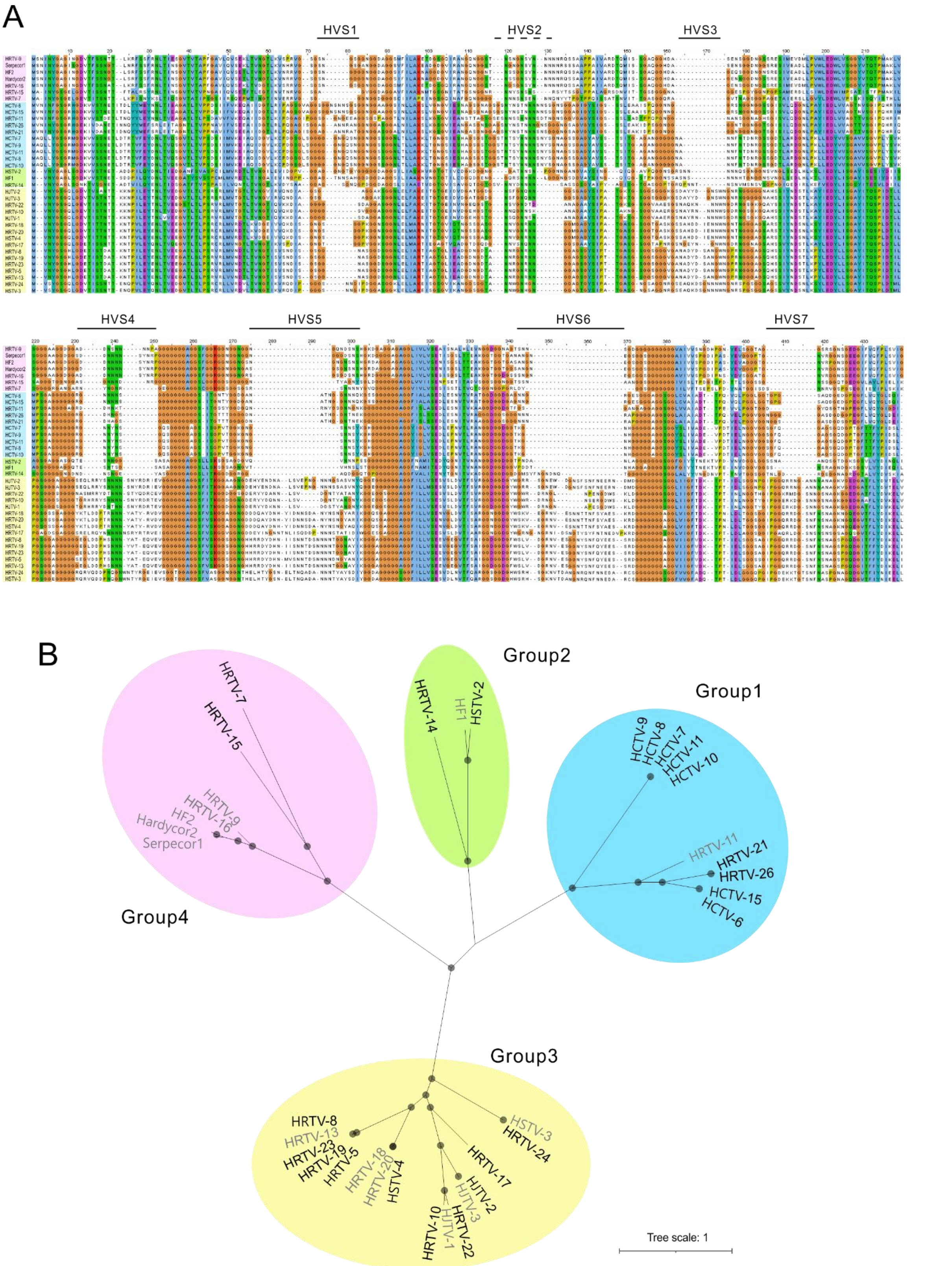

S8 Fig. The divergence of adhesin proteins encoded by viruses from the family *Hafunaviridae.* (A) Pairwise sequence alignments of adhesins encoded by viruses from the genera *Haloferacalesvirus* and *Mincapvirus*. The hypervariable segments (HVSs) which are predicted to interact with host receptor are indicated. (B) Phylogeny of the adhesins. Viruses that were used for efficiency of plating (EOP) assay in this study are indicated in black, otherwise in grey. Nodes with bootstrap support values greater than 90% are indicated with dots.
