# Supplementary material for: Diversity, taxonomy, and evolution of archaeal viruses of the class Caudoviricetes
Source: PLoS Biol. 2021 Nov 9;19(11):e3001442. doi: 10.1371/journal.pbio.3001442 (PMC8651126; doi:10.1371/journal.pbio.3001442)
Supplement: S9 Fig — Pairwise sequence alignments were produced for each of the 4 adhesin groups. The HVSs identified in S8 Fig are indicated in each group. The ratio of shared numbers of sensitive hosts/the total numbers of sensitive hosts of each 2 tested viruses was calculated (see S2 Data), and the heat map of hs versus adhesin pi was generated for each adhesin group. hs, host range similarity; HVS, hypervariable segment; pi, protein identity. (PDF) [file pbio.3001442.s020.pdf]

Group1

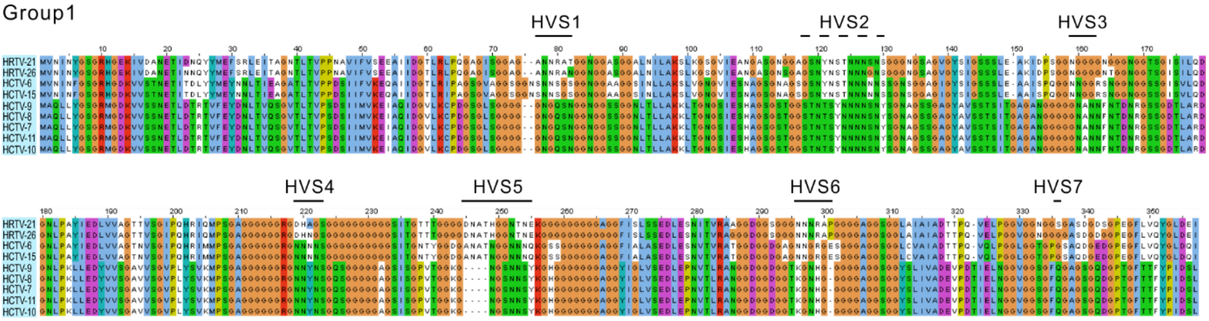

										pi
HRTV-26	100	97.2	73.8	73.8	51.5	51.5	51.5	51.5	51.5	
HRTV-21	88.9	100	73.5	73.5	52.1	52.1	52.1	52.1	52.1	
HCTV-6	54.5	63.6	100	100	56	56	56	56	56	
HCTV-15	54.5	63.6	100	100	56	56	56	56	56	
HCTV-7	46.2	53.8	66.7	66.7	100	100	100	100	100	
HCTV-9	46.2	53.8	66.7	66.7	100	100	100	100	100	
HCTV-11	46.2	53.8	66.7	66.7	100	100	100	100	100	
HCTV-8	50	58.3	58.3	58.3	90.9	90.9	90.9	100	100	
HCTV-10	50	58.3	58.3	58.3	100	100	100	100	100	
hs	HRTV-26	HRTV-21	HCTV-6	HCTV-15	HCTV-7	HCTV-9	HCTV-11	HCTV-8	HCTV-10	

0% 100%

Group2

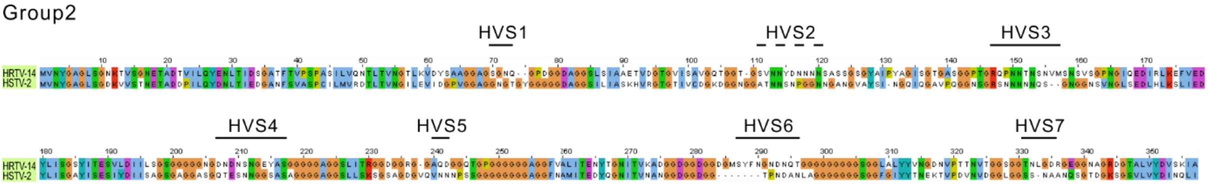

			pi
HRTV-14	100	51.4	
HSTV-2	50	100	
hs	HRTV-14	HSTV-2	

0% 100%

Group3

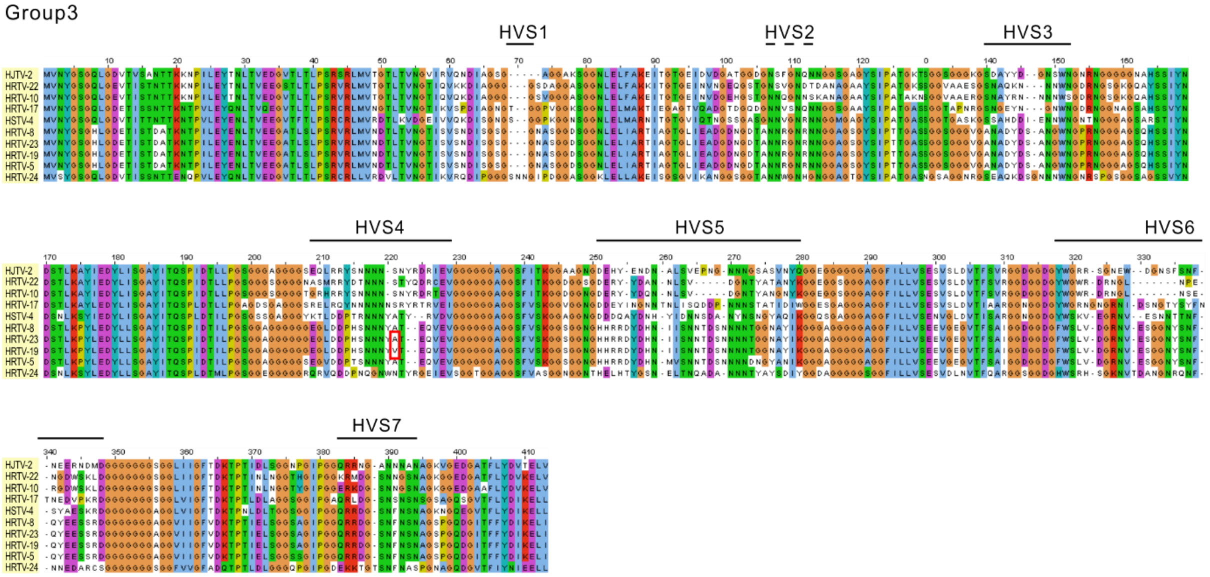

											pi
HRTV-8	100	97.5	100	99.8	69.7	59.7	60.5	62	64	64	
HRTV-5	70	100	97.5	97.3	69.9	59.9	61.4	61.7	63.5	63.8	
HRTV-23	63.6	36.4	100	99.8	69.7	59.7	60.5	62	64	64	
HRTV-19	53.8	30.8	80	100	69.4	59.7	60.5	62	63.9	64	
HSTV-4	57.1	36.7	66.7	83.3	100	60.3	62.1	60.5	63.1	68.2	
HRTV-24	38.5	36.4	60	63.6	66.7	100	55.3	56	59.2	61.4	
HRTV-22	36.4	33.3	44.4	36.4	41.7	62.5	100	89.7	73.2	64	
HRTV-10	40	29.4	45.4	50	42.8	45.4	30	100	75.1	63.9	
HJTV-2	41.2	50	27.3	33.3	38.5	27.3	22.2	15.8	100	66	
HRTV-17	36.4	33.3	30	25	30.8	30	25	18.2	22.2	100	
hs	HRTV-8	HRTV-5	HRTV-23	HRTV-19	HSTV-4	HRTV-24	HRTV-22	HRTV-10	HJTV-2	HRTV-17	

0% 100%

Group4

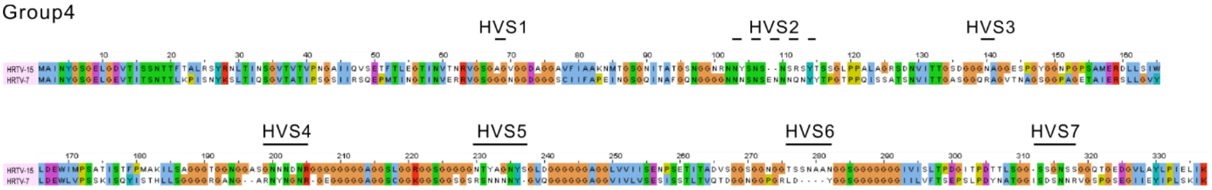

			pi
HRTV-7	100	51.9	
HRTV-15	25	100	
hs	HRTV-7	HRTV-15	

0% 100%

S9 Fig. The effect of mutations in adhesin to the host range changes in viruses of the four adhesin groups (see S8 Fig). Pairwise sequence alignments were produced for each of the four adhesin groups. The hypervariable segments (HVSs) identified in S8 Fig are indicated in each group. The ratio of shared numbers of sensitive hosts/the total numbers of sensitive hosts of each two tested viruses was calculated (see S2 Data), and the heat map of host range similarity (hs) versus adhesin protein identity (pi) was generated for each adhesin group.
