# Supplementary material for: Diversity, taxonomy, and evolution of archaeal viruses of the class Caudoviricetes
Source: PLoS Biol. 2021 Nov 9;19(11):e3001442. doi: 10.1371/journal.pbio.3001442 (PMC8651126; doi:10.1371/journal.pbio.3001442)
Supplement: S10 Fig — The same colors as in S8B Fig were used to indicate the Groups of adhesin proteins encoded by the corresponding viruses. Nodes with the bootstrap support values greater than 90% are indicated with dots. (PDF) [file pbio.3001442.s021.pdf]

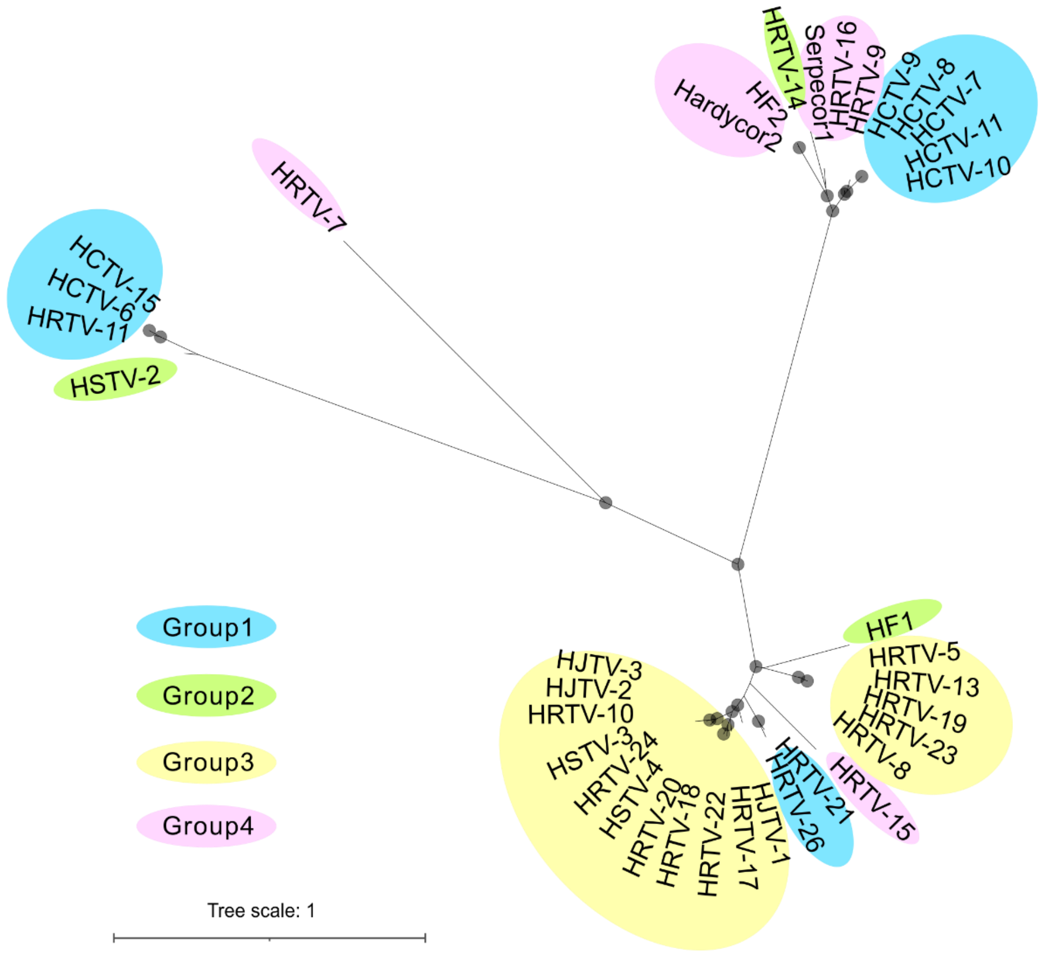

S10 Fig. Phylogenetic tree of VP1-like proteins encoded by viruses from the *Hafunaviridae.* The same colors as in S8B Fig were used to indicate the Groups of adhesin proteins encoded by the corresponding viruses. Nodes with the bootstrap support values greater than 90% are indicated with dots.
