# Supplementary material for: Diversity, taxonomy, and evolution of archaeal viruses of the class Caudoviricetes
Source: PLoS Biol. 2021 Nov 9;19(11):e3001442. doi: 10.1371/journal.pbio.3001442 (PMC8651126; doi:10.1371/journal.pbio.3001442)
Supplement: S11 Fig — The homologs retrieved from the metagenomics databases were clustered at 90% identity, and the representative of each cluster is indicated as a dot in the branch with the color denoting the source of its biome. The circle outside of the tree consisting of tiles indicates the biome compositions from the corresponding PC, which is represented by the dot in the branch. The dot in the outermost ring indicates the (predicted) virus type (colored according to its putative host) in the corresponding branch. The names of the tailed virus isolates of haloarchaea and methanogens, as well as the fosmids generated from a saltern of Santa Pola in Spain, are shown on the tree. arTV, archaeal tailed virus; PC, protein cluster. (PDF) [file pbio.3001442.s022.pdf]

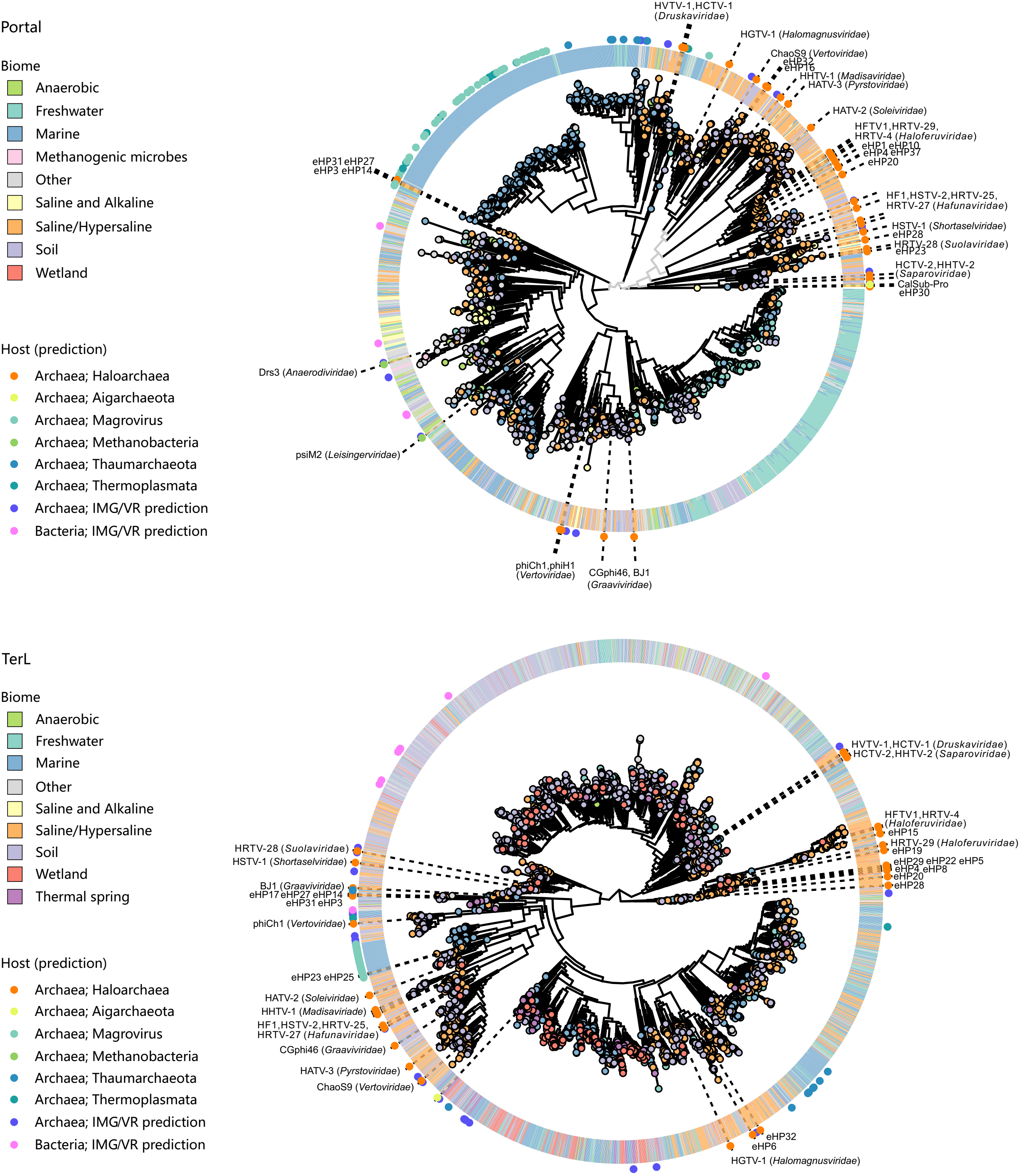

S11 Fig. Phylogenetic analysis of the portal and TerL proteins encoded by arTVs. The homologs retrieved from the metagenomics databases were clustered at 90% sequence identity, and the representative of each cluster is indicated as a dot in the branch with the color denoting the source of its biome. The circle outside of the tree consisting of tiles indicates the biome compositions from the corresponding PC, which is represented by the dot in the branch. The dot in the outermost ring indicates the (predicted) virus type (colored according to its putative host) in the corresponding branch. The names of the tailed virus isolates of haloarchaea and methanogens, as well as the fosmids generated from a saltern of Santa Pola in Spain, are shown on the tree. arTV, archaeal tailed virus; PC, protein cluster.
