# Supplementary material for: Diversity, taxonomy, and evolution of archaeal viruses of the class Caudoviricetes
Source: PLoS Biol. 2021 Nov 9;19(11):e3001442. doi: 10.1371/journal.pbio.3001442 (PMC8651126; doi:10.1371/journal.pbio.3001442)
Supplement: S5 Fig — The L21e encoded by Halorubrum californiensi, Natrinema pallidum, and Haloarcula marismortui are used as references in the alignment. arTV, archaeal tailed virus. (PDF) [file pbio.3001442.s016.pdf]

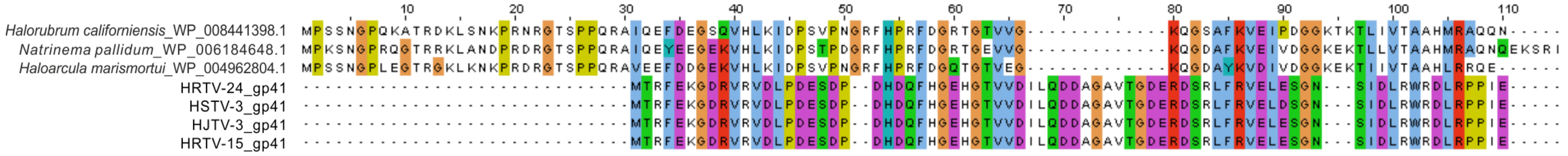

S5 Fig. Multiple sequence alignment of putative L21e homologs encoded by archaeal tailed viruses from the family *Hafunaviridae*. The L21e encoded by *Halorubrum californiensi*, *Natrinema pallidum* and *Haloarcula marismortui* are used as references in the alignment.
