# Supplementary material for: Diversity, taxonomy, and evolution of archaeal viruses of the class Caudoviricetes
Source: PLoS Biol. 2021 Nov 9;19(11):e3001442. doi: 10.1371/journal.pbio.3001442 (PMC8651126; doi:10.1371/journal.pbio.3001442)
Supplement: Attachment — Submitted filename: Responses-to-comments.pdf [file pbio.3001442.s024.pdf]

POINT-BY-POINT RESPONSES TO THE REVIEWERS' COMMENTS

Reviewer #1: The manuscript entitled ‚Diversity, taxonomy and evolution of archaeal viruses of the class Caudoviricetes' is a comprehensive analysis of 37 new genomes of archaeal tailed viruses (arTVs) including already sequenced genomes of arTVs. The classification of the arTVs into 14 new viral families within the class Caudoviricetes is proposed based on analysis with GRAViTy and vConTACT.

RESPONSE: Thank you for the positive assessment of our work and for constructive comments.

Major comments:
The advantage of isolated viruses versus viral genomes from metagenomics data, in particular the possibility to determine the host range, is nicely pointed out in the introduction. The viruses sequenced here have been isolated in previous studies and the other virus genomes discussed here have been described in other publications. Therefore, a more detailed analysis of the host range and the life cycle of the newly isolated viruses would be of interest.

Questions that would be of interest:
Do the proposed viral families correlate with a similar host range, life cycle (integration or not), sample location?

RESPONSE: The host range of viruses sequenced in this work has been experimentally tested (either previously or in this study) on a panel of haloarchaea (Figure 5, Tables S10 and S11). The availability of genome sequences now allowed us to assess the correspondence between the breadth of the host range and the newly established genome-based taxonomy (i.e., genetic distance). We did not observe obvious relationship between the host range and virus taxonomy, with some representatives of the same family displaying very broad host range and others being able to infect only one strain. Thus, host range determinants appear to be virus-specific (this is now explicitly stated in the revised text). We have investigated this more deeply on the example of hafunaviruses, the most populous family of arTVs, for which a number of genome sequences of closely related isolates are available.

In the case of *Hafunaviridae*, the host range largely depends on the variant of virus encoded tail adhesin. The adhesin encoding genes are fast evolving and frequent recombination in this region was observed among viruses from the same species as well as between viruses from different genera. As a result, there is considerable variation in the host ranges even for viruses belonging to the same species (this is discussed in the section "Mutations in tail fiber genes determine the broad host range of hafunaviruses" in the main text and associated Figure 5). It is more difficult to draw conclusion about the host range of other arTVs, because of the limited number of member in these families, with some families including only one species (e.g., HATV-2 from *Soleiviridae* and HGTV-1 from *Halomagnusviridae*).

Unlike for hyperthermophilic archaeal viruses, for most arTVs, there is no relationship between genetic closeness (i.e., members of the same taxon) and site of virus isolation. For six out of the seven viral families with more than one isolate (*Hafunaviridae, Druskaviridae, Haloferuviridae, Graaviviridae, Vertoviridae* and *Leisingerviridae*), members were isolated from two to five geographically remote locations (see column "virus origin" in Supplementary Table S1). In the cases of families *Hafunaviridae* and *Druskaviridae*, which contain members belonging to the same species, nearly identical viruses were isolated from distant locations, e.g. hafunavirus HRTV-10 was isolated from Israel, whereas HRTV-18 from Thailand; druskavirus HCTV-1 was isolated from Italy whereas HCTV-16 from Thailand, etc. Conversely, the susceptible hosts for arTVs also originate from

geographically remote sites. The only case where viruses belonging to the same family were isolated from the same sampling site is presented by HCTV-2 and HHTV-2 from *Saparoviridae* (both isolated from Samut Sakhon, Thailand).

Some of the isolated viruses encode integrases e.g. HRTV-8, HRTV-26, HRTV-27 - do they integrate? Integrases are not discussed when discussing the genomic content of viruses. Do only viruses of a particular family encode integrases?

RESPONSE: To answer this question, we added a new subsection “Integrases” (line 302-319) under the section “Gene content of archaeal tailed viruses” in the main text. All members of four viral families, namely, *Hafunaviridae* (HF1-like), *Graaviviridae* (BJ1-like), *Vertoviridae* (phiCh1-like) and *Leisingerviridae* (psiM2-like) encode integrases. To assess their integration potential, we searched the available archaeal genomes in the NCBI database for proviruses, which would be considered as members of the four families based on the established demarcation criteria. We found proviruses from all four families (new Supplementary Table S9 and Fig. S6). Consistently, viruses from *Hafunaviridae* have been observed to form either clear (e.g. HRTV-27, HRTV-13, HSTV-4, etc.) or turbid (e.g. HRTV-26, HRTV-20, HCTV-7, etc.) plaques on the cell lawns of their natural hosts (Table 2 from Atanasova et al., 2015), suggesting that viruses of this family can undergo either lysogenic or lytic life cycles, although the exact regulation remains unclear. Besides, phiCh1 has been shown to be able to integrate into host chromosome (Witte et al., 1997). Taken together, viruses from these four arTV families have the potential to integrate into the host chromosomes presumably using the encoded integrases. Notably, in the *Druskaviridae*, only one member, HCTV-5, encodes a tyrosine recombinase. However, this protein is more closely related to the invertase of phiCh1-like viruses, which has been shown to be responsible for the inversion of the tail-fiber module (Klein et al., 2012). Consistently, no HCTV-5-like proviruses could be identified in the available archaeal genomes.

Interestingly, we identified proviruses (encoding integrases) related to *Haloferuviridae* and *Anaerodiviridae* (Supplementary Table S9, Fig.S6 in this study), although none of the currently isolated members of these families encodes integrases, indicating that the integration module and hence the integration ability can be occasionally gained by arTVs. No proviruses related to viruses from the other eight families were identified, suggesting a strictly lytic life cycle for viruses from these families.

Other comment:
Lane 290-297: The fact that HGTV-1 encodes a great number of tRNA is already discussed in earlier publications. Are there any new conclusions, for example tRNAs enabling a broader host range?

RESPONSE: To answer this question, we evaluated the relationship between the number of tRNAs per genome and the determined host ranges of 13 myoviruses (including HGTV-1) from three families. The efficiency of plating of these viruses on 29 haloarchaeal strains belonging to five genera was tested previously (Table S3 from Atanasova et al., 2012). There is no obvious correlation between the number of virus-encoded tRNAs and the number of sensitive host strains (correlation r = -0.47) or between the number of viral tRNAs and the number of host genera (correlation r = 0.2) (see Figure 1 below). HGTV-1, which encodes the largest numbers of tRNAs among arTVs, did not have a broader host range than arTVs with fewer or even non tRNA genes. The analysis of codon usage of HGTV-1 versus that of its host *Halogranum* sp. SS5-1 would provide insight into the function of viral tRNAs. Unfortunately, the whole genome sequence of *Halogranum* sp. SS5-1 is currently not available.

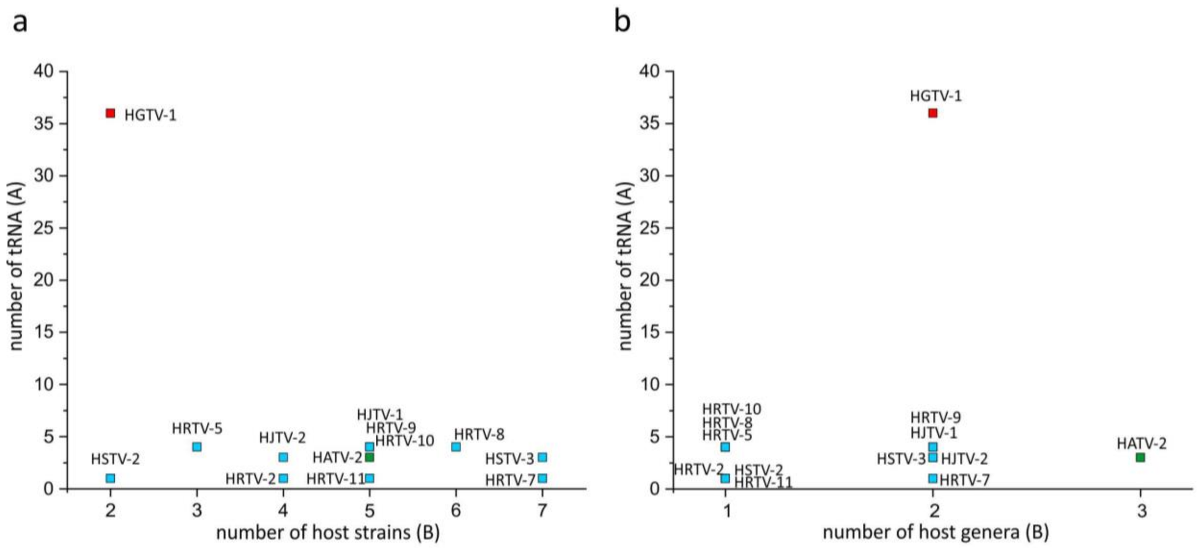

Pearson Correlations (a)

		A	B
A	Pearson Corr.	1	-0.46964
	Sig.	--	0.1054
B	Pearson Corr.	-0.46964	1
	Sig.	0.1054	--

2-tailed test of significance is used

Pearson Correlations (b)

		A	B
A	Pearson Corr.	1	0.20001
	Sig.	--	0.51238
B	Pearson Corr.	0.20001	1
	Sig.	0.51238	--

2-tailed test of significance is used

Figure 1. Relationship between numbers of viral tRNA and host ranges. (a) Relationship between numbers of viral tRNA and numbers of sensitive host strains. (b) Relationship between numbers of viral tRNA and numbers of host genera. The absolute value of Pearson correlation r >0.7 is considered strong. Viruses from different families are indicated with distinct colors.

Minor comments:
A description of the supplementary tables would be nice in the supplementary file.

RESPONSE: Added.

Supplementary table S9 Table description within the table:
Not clear: 'coloured fields have been tested' - 'empty fields have not been tested'. What about coloured empty fields - have they been tested - if yes what was the result, if not please clarify description.

RESPONSE: The colored empty fields have been tested but the infection of the corresponding strains was not observed. The description within Table S9 (now Table S10) has been modified: “Colored fields indicate that the infectivity of a virus has been tested on a particular host strain. In the case of successful infection, the efficiency of plating is indicated, whereas in the absence of infection, the field is left open”.

Supplementary table S10 - spelling mistake lane 7 ('stains' instead of 'strains')

RESPONSE: Corrected (now Table S11).

Supplementary information lane 155: 'encode' instead of 'encoding'

RESPONSE: Corrected.

Main text lane 197-198: rephrase

RESPONSE: The sentence has been rephrased into “A similar function could be envisioned for the RNA- or DNA-specific micrococcal nucleases encoded by HRTV-17, phiH1, HCTV-2 and HHTV-2”.

Reviewer #2: The paper by Lie et al. provides new and valuable insights into the diversity, function, and phylogenetic relationships among the Caudoviricetes class of viruses. The work reported here more than doubles of knowledge of these arTV. It is remarkable that 63 arTV viral genomes can lead to the proposed formation of 14 new viral families. The phylogenetic analysis is well done, compelling, and supports the formation of these 14 new families. The presented (bioinformatic) annotation of genes in these new viruses is interesting and thoughtful without being too overly speculative. The data presenting on virus isolate's host range and their correlation with tail fiber adhesion proteins well done, although in retrospect, it is not all that surprising of a finding. More interesting was the (likely) different origins of some of the arTV viral families and their distinct separation from tailed bacteriophages. Overall, this is an excellent manuscript.

RESPONSE: Thank you for the positive assessment of our work and for constructive suggestions.

I have only minor suggestions for improving this manuscript. They include the following.

1. I would tone down a bit how robust the taxonomic framework is (i.e. lines 99-101) given that some families are represented by only a single member. As the author's themselves state, there is likely much more diversity out there in this class of viruses (lines 424-425).

RESPONSE: We agree with the reviewer. The statement about the robustness of the taxonomic framework has been toned down (line 99-101): “Collectively, our results provide the first global overview of arTV diversity and evolution and establish a taxonomic framework for their classification.”

2. It would be useful if the authors provide more details and discussion of the arTV MCP and portal proteins. How distinct or not are the secondary structures of the 9 MCP clades. Likewise for the portal proteins. Are the two trees coherent with each other or not?

RESPONSE: To address this question we performed phylogenetic congruence analysis and compared the MCP and portal trees (new Fig. S12). Generally, arTVs formed the same clades in the two trees, suggesting that portal and MCP proteins coevolved in arTVs and are rarely separated by recombination. The possible exceptions are presented by HGTV-1 and ChaoS9, with the latter being notoriously chimeric (PMID: 30832293; see also Fig. S1G). With regard to the comparison of the secondary structures, we performed structural modeling using AlphaFold2 and RoseTTAFold for representatives of all 14 arTV families as well as selected uncultured arTVs. The structural models were then compared to each other in all-against-all analysis and a cladogram was derived from pairwise structural similarity (Z) scores (new Fig. 7). This analysis confirmed that all identified MCPs have the HK97 structural fold, shared with tailed bacteriophages and eukaryotic herpesviruses, and revealed the same 9 MCP clades obtained using sequence-based phylogenetic analysis. Besides the subtle differences throughout the protein in different members, the more pronounced variation was present in the N-termini of the MCP. In particular, some arTVs (e.g., HATV3, HCTV2, HFTV1, HVTV1, etc) contained N-terminal 100-120 aa extensions, equivalent to the scaffolding delta domain of the HK97 MCP, which is essential for capsid assembly and is cleaved from the mature MCP. These results are also described in the Supplementary text.

3. The authors could also include a brief discussion of how these arTVs are related or not related to other non-arTV archaeal viruses.

RESPONSE: The arTVs are disconnected from all non-tailed archaeal viruses in the network analysis using vConTACT v2.0. Only a few genes are shared, including those encoding a methyltransferase, transposase, adenylyltransferase, AAA+ domain protein and proteins of unknown function (see Table S4 PC(protein cluster)_07022, PC_02906, PC_00627, PC_14592, PC_04806, PC_07000, PC_13203 and PC_13880). We have modified the sentence (line 131-133): "Consistent with the GRAViTy results, the network analysis revealed two assemblages of arTVs, which were disconnected from all known bacterial and non-tailed archaeal viruses. Notably, only a few genes were shared between arTVs and non-tailed archaeal viruses, including those encoding a methyltransferase, transposase, adenylyltransferase, AAA+ domain protein and proteins of unknown function (Table S4)."

References

Atanasova NS, Roine E, Oren A, Bamford DH, Oksanen HM. Global network of specific virus-host interactions in hypersaline environments. Environ Microbiol. 2012;14(2):426-40. doi: 10.1111/j.1462-2920.2011.02603.x. PubMed PMID: 22003883.

Atanasova NS, Demina TA, Buivydas A, Bamford DH, Oksanen HM. Archaeal viruses multiply: temporal screening in a solar saltern. Viruses. 2015;7(4):1902-26. doi: 10.3390/v7041902. PubMed PMID: 25866903.

Witte A, Baranyi U, Klein R, Sulzner M, Luo C, Wanner G, Krüger DH, Lubitz W. Characterization of Natronobacterium magadii phage ϕCh1, a unique archaeal phage containing DNA and RNA. Mol Microbiol. 1997;23(3):603-616. doi: 10.1046/j.1365-2958.1997.d01-1879.x. PubMed PMID: 9044293.

Klein R, Rössler N, Iro M, Scholz H, Witte A. Haloarchaeal myovirus ϕCh1 harbours a phase variation system for the production of protein variants with distinct cell surface adhesion specificities. Mol Microbiol. 2012;83(1):137-150. doi: 10.1111/j.1365-2958.2011.07921.x. PubMed PMID: 22111759.
