# Supplementary material for: Diversity, taxonomy, and evolution of archaeal viruses of the class Caudoviricetes
Source: PLoS Biol. 2021 Nov 9;19(11):e3001442. doi: 10.1371/journal.pbio.3001442 (PMC8651126; doi:10.1371/journal.pbio.3001442)
Supplement: S12 Fig — The best-fitting models identified for the MCP and portal trees were selected by PhyML and were Blosum62+G+I+F and LG+G+I+F, respectively. Same colors are used for clades containing the same group of viral members in both trees. HGTV-1 and related haloarchaeal virus fosmids, as well as ChaoS9, that clustered differently in the 2 trees are indicated by lines. Bootstrap values are shown next to the nodes, with values above 80 shown in black and those below 80 in gray. arTV, archaeal tailed virus; MCP, major capsid protein. (PDF) [file pbio.3001442.s023.pdf]

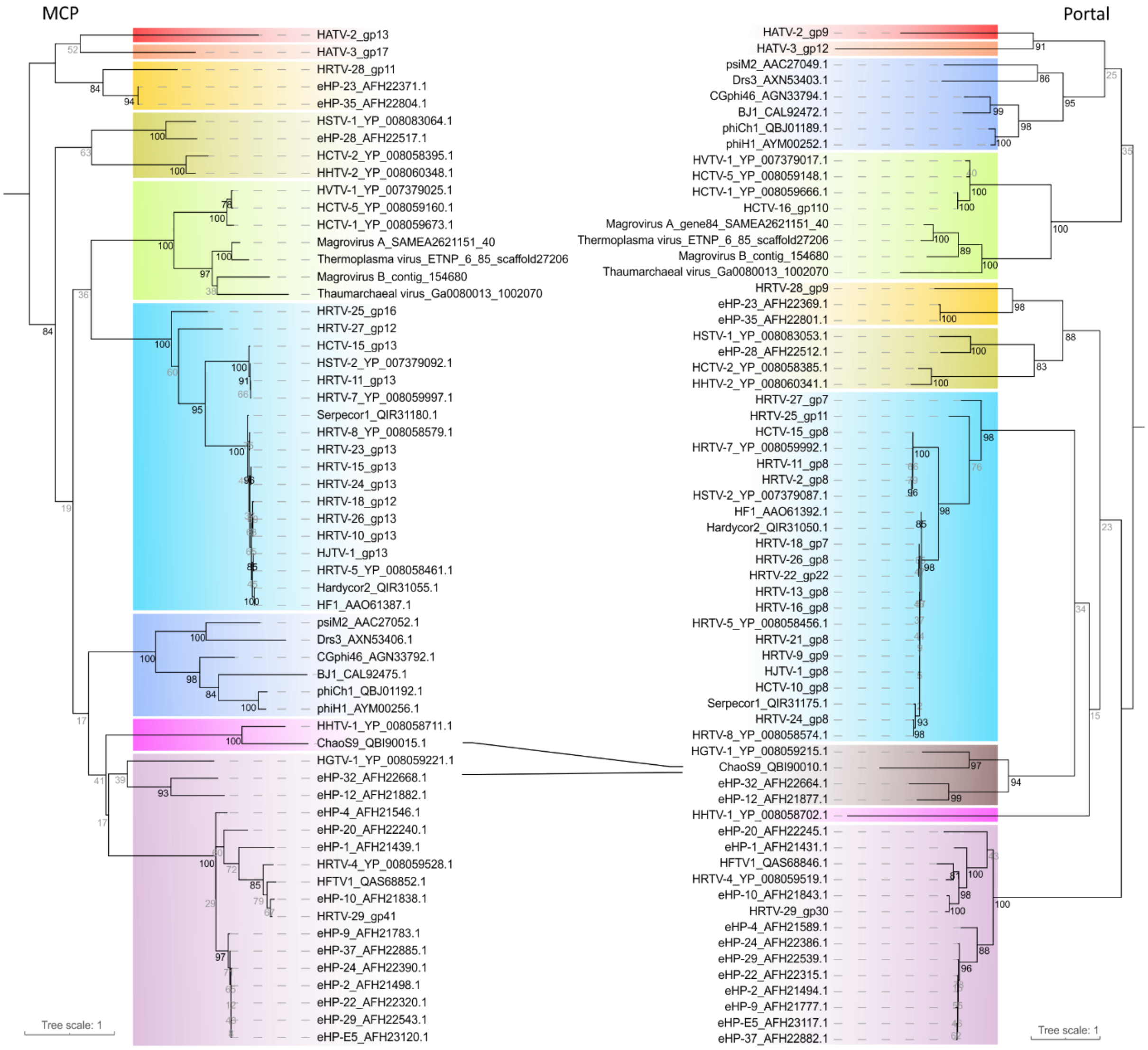


S12 Fig. Maximum likelihood phylogenetic trees of the arTVs MCPs and portal proteins. The best-fitting models identified for the MCP and portal trees were selected by PhyML and were Blosum62+G+I+F and LG+G+I+F, respectively. Same colors are used for clades containing the same group of viral members in both trees. HGTV-1 and related haloarchaeal virus fosmids, as well as ChaoS9, that clustered differently in the two trees are indicated by lines. Bootstrap values are shown next to the nodes, with values above 80 shown in black and those below 80 in grey.
